# Regulation of fatty acid composition related to ontogenetic changes and niche differentiation of a common aquatic consumer

**DOI:** 10.1007/s00442-020-04668-y

**Published:** 2020-05-21

**Authors:** F. Chaguaceda, P. Eklöv, K. Scharnweber

**Affiliations:** Department of Ecology and Genetics; Limnology, Uppsala University, Uppsala, Sweden

**Keywords:** Food-web, Life-history trade-offs, Ontogenetic shifts, *Perca fluviatilis*, Stable isotopes

## Abstract

Fatty acids (FAs) are key nutrients for fitness which take part in multiple physiological processes over the ontogeny of organisms. Yet, we lack evidence on how FA nutrition mediates life-history trade-offs and ontogenetic niche shifts in natural populations. In a field study, we analyzed ontogenetic changes in the FAs of Eurasian perch (*Perca fluviatilis* L.), a widespread fish that goes through ontogenetic niche shifts and can have high individual niche specialization. Diet explained most of the variation in the FA composition of perch dorsal muscle over early ontogeny (28%), while the total length explained 23%, suggesting that perch significantly regulated FA composition over early ontogeny. Condition explained 1% of the remaining variation. 18:3n-3 (ALA) and 18:4n-3 (SDA) indicated planktivory; 18:1n-7, benthivory; and 22:6n-3 (DHA), piscivory in perch diet. Conversely, perch regulated long-chained polyunsaturated fatty acids (PUFAs), such as 20:5n-3 (EPA), 20:4n-6 (ARA) and 22:6n-3 (DHA) over ontogeny, emphasizing the role of such FAs in early growth and sexual maturation. Adult perch increasingly retained 16:1n-7 and 18:1n-9 suggesting higher energy storage in older perch. Furthermore, differences in DHA availability in diet correlated with intra-cohort differences in perch growth, potentially hindering the overall use of benthic resources and promoting earlier shifts to piscivory in littoral habitats. Overall, this study indicates that in addition to diet, internal regulation may be more important for FA composition than previously thought. Differences between FA needs and FA availability may lead to life-history trade-offs that affect the ecology of consumers, including their niche.

**Electronic supplementary material:**

The online version of this article (10.1007/s00442-020-04668-y) contains supplementary material, which is available to authorized users.

## Introduction

Organisms often undergo life-history trade-offs in which they compromise the expression of different traits in order to maximize fitness (Stearns 1992). Changes in selective pressures over ontogeny affect life-history trade-offs, and may lead to shifts in habitat use or specialization of consumers on a particular resource, going through ontogenetic niche shifts (Werner and Gilliam 1984). As organisms grow, they need to deal with exponential increases of energetic demands due to allometric scaling of metabolism (Kleiber 1932; Peters 1983). On the other hand, increasing body size allows for consumption of larger, more energy rich prey (e.g. Werner and Gilliam 1984; Mittelbach and Persson 1998) and the access to a broader prey size range (Wilson 1975), while decreasing or increasing predation risk from different size-selective predators (De Roos and Persson 2013). Although life-history trade-offs and their implications for ontogenetic niche shifts have been extensively studied (e.g. Werner and Gilliam 1984; Mittelbach and Persson 1998) few studies have investigated the role of nutrients in such processes (but see Maazouzi et al. 2011; Vrede et al. 2011; Showalter et al. 2016). This is particularly important, as the nutritional composition of food is a well-known driver of fitness (Brett and Müller-Navarra 1997; Sterner and Elser 2002; Raubenheimer et al. 2009), often more important than mere resource quantity (e.g. Brett et al. 2009; Twining et al. 2016b). To understand life-history trade-offs and ontogenetic niche shifts in nature, it is therefore necessary to incorporate food chemical composition into the framework, taking a closer look at nutrient allocation and nutrient regulation over ontogeny and whether those have measurable implications for consumer fitness.

Fatty acids (FAs) are critical biochemical compounds for organisms, being a major component of membranes, energy reserves and hormonal regulation (Tocher 2003). Among them, long-chained polyunsaturated fatty acids (PUFAs), such as EPA (20:5n-3), ARA (20:4n-6) and DHA (22:6n-3) are particularly important for the growth and reproduction of consumers across terrestrial and aquatic systems (Yu and Sinnhuber 1979; Brett et al. 2009; Twining et al. 2016a). However, vertebrate animals like fish cannot synthesize PUFAs de novo (Bell and Tocher 2009), and may have limited abilities to synthesize long-chained PUFAs from other PUFA compounds (Tocher 2003), making direct consumption of long-chained PUFAs highly necessary and advantageous for consumers.

Yet, the availability of PUFAs in food differ substantially among prey taxa from different habitats (Lau et al. 2012; Hixson et al. 2015). For instance, aquatic algae produce much higher amounts of long-chained PUFAs than terrestrial plants, leading to fundamental differences in the availability of PUFAs between aquatic and terrestrial food webs (e.g. Hixson et al. 2015; Ruess and Müller-Navarra 2019). Furthermore, organisms at higher trophic levels tend to have higher proportions of long-chained PUFAs (Strandberg et al. 2015; Twining et al. 2016a). Therefore, changes in the habitat use and trophic level in consumers in response to ontogenetic niche shifts will likely affect the ingestion of PUFAs and their potential effects on the fitness of consumers.

Furthermore, the requirements for different FAs are likely to be affected by changes in life-history processes (e.g. Tocher 2010). High amounts of omega-3 PUFAs, such as DHA are necessary for growth and tissue differentiation (Ballantyne et al. 2003; Tocher 2010). ARA and EPA are also in high demand as precursors of eicosanoids, which regulate multiple physiological processes over ontogeny such as immune response, neural function and reproduction (Tocher 2003). Once reaching maturity, DHA and ARA have been associated with the formation and maturation of gonads (Tocher 2003), subsequently affecting reproductive success and survival of the offspring (Henrotte et al. 2010). As a result, changes in the availability and the requirements for fatty acids over the ontogeny of consumers make fatty acids good targets to study the effects of nutrients on their life-history trade-offs and ontogenetic niche shifts.

In relation to FA requirements, consumers may regulate FA composition to sustain a certain degree of homeostasis (Iverson 2009). For instance, physiologically important PUFAs are selectively retained under limiting dietary inputs, while other FAs are used for metabolism (e.g. Abi-Ayad et al. 2000; Taipale et al. 2011). Similarly, certain FAs may be selectively retained or mobilized depending on ontogenetic changes in FA needs (Henderson and Tocher 1987; Tocher 2003). However, it is not well known to which extent dietary and internal regulatory processes affect FA composition over ontogeny. This will probably affect the traditional use of FAs as trophic markers, since physiological changes that affect FA homeostasis may correlate with changes in FA composition of its dietary sources. Solving this dilemma is necessary to understand the extent to which FAs are potential causes or mere consequences of ontogenetic diet shifts in nature.

In a field study, we assessed the changes in FA composition and growth over the ontogeny of Eurasian perch, *Perca fluviatilis* L., hereafter perch, a common and widespread freshwater fish which goes through ontogenetic diet shifts, as most vertebrate predators that grow several orders of magnitude in body size (Werner and Gilliam 1984; Mittelbach and Persson 1998). As juveniles, perch feed on zooplankton, shift to benthic macroinvertebrates and as adults they feed primarily on fish (Persson 1988). Like other species showing intraspecific niche partitioning (Skúlason and Smith 1995; Bolnick et al. 2003), perch individuals often differ substantially in their diet and habitat use (Svanbäck and Eklöv 2002), increasing their niche specialization at intermediate ontogenetic stages (Svanbäck et al. 2015). Furthermore, of the resources used by perch, zooplankton provides higher proportions of omega-3 PUFAs than benthic macroinvertebrates, in which DHA is nearly absent (Lau et al. 2012; Scharnweber et al. 2016), while fish provide the highest amount of PUFAs, especially DHA (Lau et al. 2012; Kainz et al. 2017). Differences in FA allocation among different diets and high niche variation of perch over ontogeny make perch a particularly good model organism to study the factors affecting the FA composition of consumers over ontogeny and the role in FA nutrition in life-history trade-offs.

In this study, we hypothesized that: (1) FA composition of perch is linked to the FA composition of their diet, reflecting ontogenetic niche shifts as well as differences in individual resource specialization. We therefore predicted that abundant fatty acids in diet would also be more abundant in the consumer, reinforcing the role of FAs as trophic biomarkers. (2) FA composition of perch would change with body size irrespective of dietary inputs, indicating trends and shifts in FA regulation during ontogeny. Due to the high physiological importance of long-chained PUFAs for life-history processes, we expect that such fatty acids would be selectively retained in tissues as compared to other less important fatty acids. Finally, (3) we hypothesized that mismatches between dietary FA and FA requirements over ontogeny may affect life-history trade-offs in perch, and eventually promote ontogenetic niche shifts, for instance by promoting shifts to a better-quality food at the expense of less foraging efficiency or higher predation risk. These results may be applicable to other organisms with ontogenetic niche shifts and with changes in physiological demands for PUFAs over ontogeny, which are likely to be the norm rather than the exception in nature (Werner and Gilliam 1984; Twining et al. 2016a).

## Materials and methods

### Field sampling

**Fig. 1:**
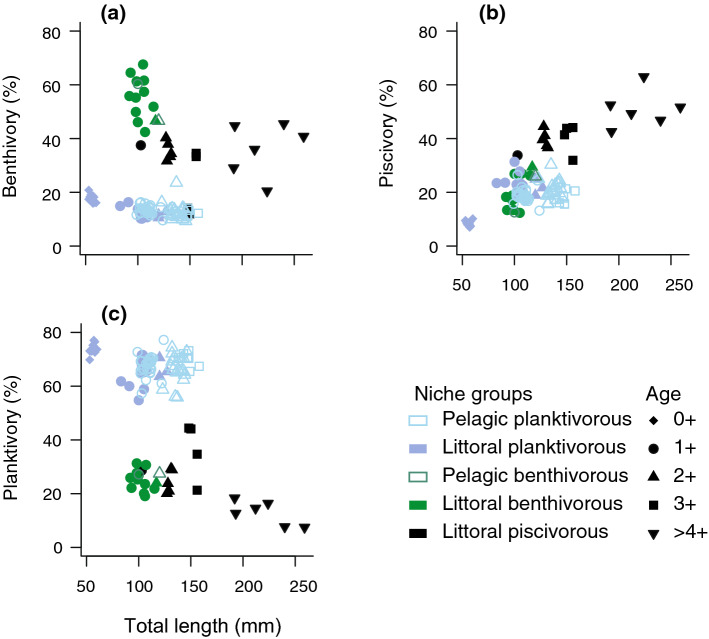
Resource use over the ontogeny of the different niche groups of perch (*Perca fluviatilis* L.) based on the stable isotope analysis. **a** Percent of benthivory; **b** piscivory and **c** planktivory of perch individuals in relation to their total length

The study was performed in the lake Erken, a mesotrophic lake located in central Sweden (59°50′N, 18°33′E) where perch exhibits resource polymorphism with respect to littoral and pelagic habitats (Svanbäck et al. 2008; Scharnweber et al. 2016; Bartels et al. 2016). Perch and their potential fish prey (ruffe, *Gymnocephalus cernua* L.; roach, *Rutilus rutilus* L.) were captured in 18 Aug 2015 (in the middle of the growing season 8 months before reproduction) using multimesh gill nets (littoral nets 30 × 1.5 m; pelagic nets 27.5 × 6 m). We set two pelagic nets in the open water environment and two littoral nets in shallow, near-shore areas for 12 h overnight. To minimize post mortem degradation of FAs, we selected only fish with clear red gills and froze them at −20 °C for up to 37 days. In the laboratory, perch and prey fish were partially thawed. We measured the total length (*L*_T_) to the nearest millimeter, and wet weight (*W*) of perch to the nearest gram, to calculate perch condition factor: $K = 100 \times (W/L_{\text{T}}^{3} ),$ according to Fulton (1904). In the end, we included 113 perch in the study (age 0+ to 6+; total length 53–259 mm); 57 from the pelagic and 56 from the littoral zone. Age 0+ and older than 4+ individuals were only found in the littoral zone, while intermediate ontogenetic stages were found in both pelagic and littoral zones (Fig. 1).

We dissected two pieces of the dorsal muscle of each perch; one was stored at −20 °C for FA analysis; the other was dried in the oven for 24 h at 60 °C for stable isotope analysis. We used the opercular bones for age analysis (Le Cren 1947). To investigate ontogenetic trajectories in each individual, we interpolated length at age from regressions between total length and opercular diameter for littoral and pelagic perch separately using the Fraser–Lee method (Fraser 1916; Lee 1920; Francis 1990). Then we estimated growth at age (*g*) as the increment of total length between two consecutive years (*t*, *t* + 1) for each habitat, $g = \ln L_{t} - \ln L_{t + 1} ,$ according to Heibo et al. (2005).

On the same day as fish sampling, we collected zooplankton prey by multiple vertical hauls using a 100 µm mesh-size zooplankton net and littoral macroinvertebrates prey either with a sweep net (2 mm mesh size) or manually from different littoral substrates. Subsequently, we separated them into major taxonomic groups (Cladocera, Copepoda, Chironomidae, Gastropoda and Isopoda), using a dissecting microscope if needed and dried in the oven at 60 °C for 24 h for stable isotope analysis. Fatty acid data of invertebrate prey were obtained from Scharnweber et al. (2016) (see “Fatty acid analysis”).

### Stable isotope analysis

The stable isotope analysis is based on the consumers integrating a proportional mixture of isotopic values of their prey, with a certain degree of modification termed fractionation (Post 2002). For this study, we used carbon (C) and nitrogen (N) isotopic values as δ^13^C differs between benthic and pelagic production and δ^15^N increases with trophic position (Post 2002).

Oven-dried samples were ground into fine powder using a ceramic mortar and a pestle, from which we weighed approximately 1.00 mg into tin capsules, and subsequently sent it to the University of California, Davis Stable Isotope Facility, California, USA where C and N elemental and stable isotope analyses were performed, using a PDZ Europa ANCA-GSL elemental analyzer coupled to a PDZ Europa 20–20 isotope ratio mass spectrometer (Sercon, Cheshire, UK). As C/N was low (3.38 ± 0.48; mean ± SD), no lipid normalization was performed (Kiljunen et al. 2006). Isotopic values are presented in δ notation based on the ratios of each sample to a reference standard. The analysis was duplicated for 30% of the samples and the analytical error was smaller than 0.15‰ for both elements.

To estimate the long-term contribution of the different resources to the biomass of individual perch, we analyzed the δ^13^C and δ^15^N values using Bayesian mixing models in MixSIAR version 3.1.10 (Stock and Semmens 2016) using perch individual as a fixed factor nested within age. Copepods, cladocerans, benthic macroinvertebrates and fish prey were used as end-members in the model, correcting for isotopic trophic fractionation, using a fractionation factor of 0.4 ± 1.3 for δ^13^C and 3.4 ± 1.0 for δ^15^N based on Post (2002). We chose such robust and conservative estimations of fractionation factors due to the lack of direct experimental knowledge of the species at different ontogenetic stages and diets. Before running the mixing model, we carefully validated resource isotopic values and mixing geometry were from isotopic biplots and subsequently compared with the model output (Supplementary Material 1, Fig. S2).

To facilitate further analysis and visualization, we classified perch according to habitat use (i.e. pelagic and littoral) and on the main three diets over the ontogeny of perch (i.e. planktivorous, benthivorous and piscivorous perch). Perch tend to have high habitat fidelity (Eklöv 1997) and therefore we assumed that perch habitat use when captured was a meaningful measure of their long-term habitat use. Grouping according to diet was performed by k-means clustering, based on individual dietary proportions of zooplankton, macroinvertebrates and fish in diet obtained from MixSIAR models (Supplementary Material 1). We assessed the validity of these diet groups in the isospace biplot (Supplementary Material 1, Fig. S2). As a result, five niche groups of perch were obtained: littoral benthivorous (*n* = 12), littoral planktivorous (*n* = 28), pelagic planktivorous (*n* = 55), pelagic benthivorous (*n* = 2) and littoral piscivorous (*n* = 16) perch. For more information about the mixing model and cluster analysis, please see Supplementary Material 1.

### Fatty acid analysis

We used fatty acid analysis to assess qualitative diet preferences complementary to stable isotope analysis, and to investigate nutritional processes over ontogeny of perch in the different habitats. For perch and fish prey, we took a subsample of approximately 100–200 mg of tissue from the centre part of each dorsal muscle sample. Lipid extraction and FA transmethylation was performed according to Scharnweber et al. (2016), using (2/1) chloroform/methanol as organic solvent and H_2_SO_4_ as a catalyst and FA methyl esters (FAMEs) were analyzed by gas chromatography. We calculated single FAMEs concentration using a calibration curve from standard solutions of known lipid FAME mixtures of 20 fatty acids commonly known to occur in fish muscle tissues (GLC Reference standard 68 D, Nu-Chek Prep) on the area under each FA peak in the chromatogram. We inferred FA composition of zooplankton and macroinvertebrates based published data from Scharnweber et al. (2016), who analyzed samples 1 year before in the same month, from the same lake and in similar locations as the samples taken in 2015. For more information about FA analysis, see Supplementary Material 2.

### Statistical analysis

Multivariate statistical analyses were performed using PRIMER version 7.0.11 (Primer E, Plymouth, UK) with the PERMANOVA add-on. Proportions of FAs were arcsine-square-root-transformed and all ordination and tests were based on Euclidean distance matrices. We used distance-based linear modelling (DistLM) to evaluate how much of the variation in perch FA composition was explained by changes in diet, body condition and internal ontogenetic changes. As total length is a common and relevant ontogenetic measure in fish (e.g. Werner and Gilliam 1984; De Roos and Persson 2013), we used the total length as a proxy for ontogenetic change. We included the following explanatory variables in the analysis: total length on the date of capture, percent of benthivory, planktivory and piscivory in diet and condition factor. In the case of high correlations, *r* > 0.9 among explanatory variables, only one of them was included in the model.

To avoid problems of collinearity between age and diet and to maximize ranges of diet in the model, we only included 1+ to 3+ years old perch in this analysis. *P* values were determined after 9999 permutations, after stepwise selection of explanatory variables using *R*^2^ as selection criterion.

To test whether FA composition differed between habitats, we ran a one-way PERMANOVA, including habitat as fixed factor and using a Type III sum of squares (9999 permutations). We tested the effect of habitat irrespective of main explanatory variables by including habitat as a fixed factor and significant explanatory variables from DistLM analysis as covariates. In the latter model, significance was determined by restricted permutation of residuals under a reduced model (9999 permutations), using type I sum of squares (Anderson et al. 2008).

We performed similarity percentages routine (SIMPER) for pairwise comparisons between benthivorous vs. planktivorous, piscivorous vs. non-piscivorous and consecutive age groups of perch. To avoid age dependence on diet comparisons and diet dependence on age group comparisons, we used a two-way crossed design, because it summarizes dissimilarities nested within each age or niche group, respectively (Clarke et al. 2014). To estimate the importance of single FAs to overall FAs changes due to ontogeny or diet, we calculated the mean percentage contribution of all pairwise comparisons for each FA indicator.

The variation in FA composition of perch from different habitats, feeding groups and age was visualized by non-metric multidimensional scaling (nMDS) (Anderson et al. 2008).

Quadratic or linear responses of single FAs over the ontogeny of perch were analyzed using linear models of the different niche groups separately. To compare the mean differences in growth, length at age, total length, weight or condition factor among niche groups, we used non-parametric tests integrated in the R-package FSA (Ogle 2017). Statistical analysis other than multivariate analysis was performed in R software, version 3.6.1 (R Core Team 2019).

## Results

### The ontogenetic pattern of diet composition

Stable isotope mixing models revealed that perch went through ontogenetic niche shifts and showed different resource use in the different habitats (Fig. 1). Age 0+ perch were mostly planktivorous, feeding mainly on cladoceran prey (Fig. 1, Supplementary Material 1, Fig. S2, Table S7). Intermediate sizes and ages of perch showed the highest niche diversification: in the pelagic zone, perch fed mostly on zooplankton, while, in the littoral zone, both planktivorous, benthivorous and piscivorous perch were found (Fig. 1). The transition to piscivory among littoral individuals began in age 2+, where 55% were classified as piscivorous [total length (mean ± SD): 129 ± 2 mm; % piscivory (mean ± SD: 40 ± 3%)], whereas no piscivorous individuals were found in the pelagic zone (Fig. 1).

### FA composition over ontogeny

**Fig. 2:**
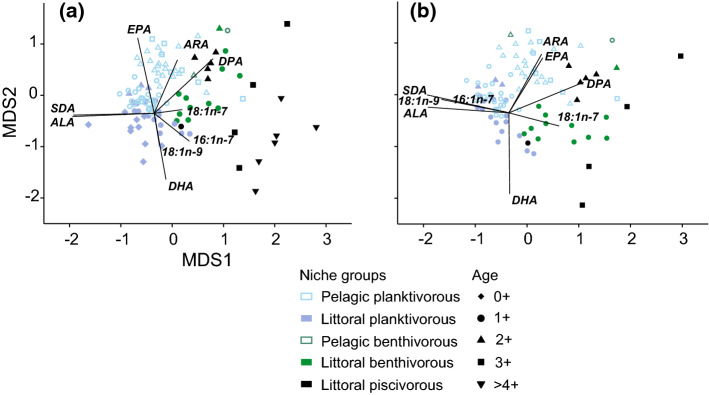
Non-metric multidimensional scaling (nMDS) representing variation the FA composition of perch over ontogeny: **a** representation of the whole dataset, *n* = 113; **b** only age classes 1+ to 3+ of perch are included, *n* = 97. Vectors represent correlations of the main FA indicators of ontogenetic and diet changes. 2D stress was 0.14 for both plots

**Fig. 3:**
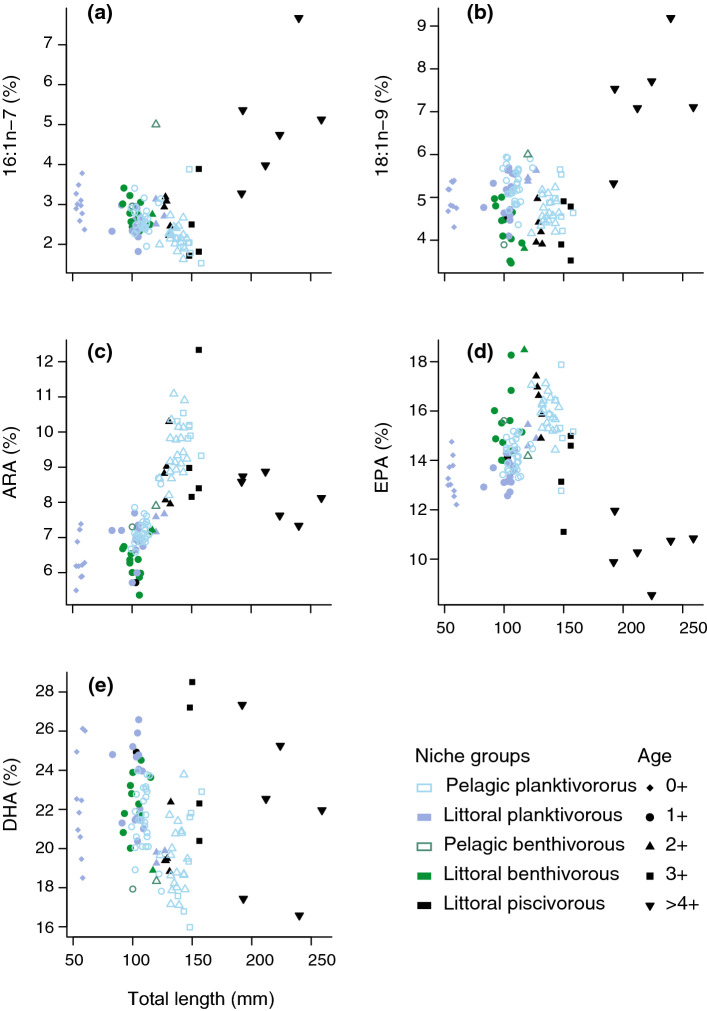
Changes in proportions of the five most responsive fatty acids over the ontogeny of perch indicated by increasing the total length **a** palmitoleic acid, 16:1n-7; **b** oleic acid, 18:1n-9; **c** arachidonic acid (ARA), 20:4n-6; **d** eicosapentaenoic acid (EPA), 20:5n-3 and **e** docosahexaenoic acid (DHA), 22:6n-3. Regression models for the ontogenetic responses of different niche groups of perch are shown in Table S3

Perch underwent significant changes in FA composition over ontogeny in relation to diet shifts and intrinsic ontogenetic changes, with a major shift in ontogenetic FA trends during year 3 (Figs. 2, 3). Sequential DistLM model for 1+ to 3+ years old perch (*R*^2^ = 0.52) revealed that the total length, diet and perch condition factors were significantly related to changes in FA composition of perch (Table 1).

**Table 1:** Influence of covariates representing diet (% planktivory, % piscivory in diet), ontogenetic change (total length) and fitness (condition factor) on the FA composition of perch at the age 1+ to 3+ using DistLM

Sequential DistLM *R*^2^ = 0.517				
Variable	Residuals *d.f.*	Pseudo-*F*	*P*	% of total variation explained
% Planktivory	95	28.25	< 0.001	22.9
Total length	94	39.58	< 0.001	22.8
% Piscivory	93	8.99	< 0.001	4.8
Condition factor	92	2.32	0.038	1.2

Covariates were tested for collinearity and sequentially included based on the *R*^2^ as selecting factor

Planktivory, which was correlated negatively with benthivory (*r* = − 0.92), explained 23% of FA variation, while piscivory explained an extra 5%, meaning that overall dietary choices explained 28% of FA variation in 1+ to 3+ years old perch (Table 1). The total length explained 23% of the variation in FA composition, while perch condition factor explained 1.2% of the remaining variation. FA composition differed significantly between littoral and pelagic perch 1+ to 3+ years old (PERMANOVA, pseudo-*F *= 7.536, *P *< 0.001; Supplementary Material 2, Table S4a). However, once the total length, diet and condition factor were included as co-variables, habitat did not have a significant effect on FA composition (PERMANOVA, pseudo-*F* = 1.875, *P *= 0.078; Supplementary Material 2, Table S4b).

According to SIMPER analysis, five FAs explained 49.1% of the dissimilarities due to diet (SDA: 18:2n-3, 17.1%; DHA: 22:6n-3, 9.4%; VA: 18:1n-7, 7.6%; ALA: 18:3n-3, 7.6%; DPA: 22:5n-3, 7.4%; Supplementary Material 2, Table S1). Planktivorous perch had higher SDA and ALA and lower DPA and 18:1n-7 than benthivorous perch (Fig. 2b, Supplementary Material 2, Table S1). Piscivorous perch had lower proportions of SDA, ALA and higher proportions of DHA and 18:1n-7 (Fig. 2b, Supplementary Material 2, Table S1).

Five FAs explained 54.5% of the cumulative differences of FA composition over ontogeny (DHA: 22:6n-3, 25.7%; POA: 16:1n-7, 11.3%; EPA: 20:5n-3, 7.4%; OA: 18:1n-9 5.8%; ARA: 20:4n-6, 5.6%; Supplementary Material 2, Table S2). Overall, FA composition of young-of-the-year perch had high DHA, intermediate EPA and low ARA proportions (Fig. 3). After age 1+, DHA proportions in non-piscivorous perch began to decrease with body size, while EPA and ARA increased until reaching age 3+ and 150 mm total length (Figs. 2b, 3, Table S3). At age 3+, a major shift in FA trends occurred (pairwise PERMANOVA among 3+ piscivorous, 3+ pelagic planktivorous and > 3+ piscivorous perch, *t *> 2.26, *P* < 0.05; Fig. 2a) after which, EPA proportions decreased linearly with the total length, while proportions of 16:1n-7 and 18:1n-9 increased in piscivorous perch (Fig. 3, Table S3). The sum of all FA concentrations decreased until age 3 + perch with no trends for older perch (Supplementary Material 2, Fig. S4).

### Growth and length relationships of perch

Back calculation of length at age revealed that after the first growing season, during which perch reached 75 ± 9 mm, the total length of perch increased linearly, around 30 mm per year (Supplementary Material 3, Fig. S5, Table S7). Age 1+ and age 2+ pelagic planktivorous perch were the longest and the heaviest of their cohort, which was also extrapolated to the previous two growing seasons based on the length at age calculations (Dunn’s test on Kruskal–Wallis multiple comparison, *P*-adjusted < 0.05; Supplementary Material 3, Tables S4 and S5). However, these differences did not occur in age 3+ perch (Kruskal–Wallis test, *P* = 0.13; Supplementary Material 3, Table S5), as piscivorous perch had higher growth than pelagic planktivorous perch during their third growing season (Kruskal–Wallis test, *P* = 0.01; Supplementary Material 3, Fig. S5, Table S6). The perch groups did not differ in condition factor (Dunn’s test on Kruskal–Wallis multiple comparison, *P*-adjusted < 0.05; Supplementary Material 3, Table S5). The only significant inter-cohort differences within the niche groups was the higher first year growth of pelagic planktivorous perch during 2014 (Dunn’s test on Kruskal–Wallis multiple comparison, *P*-adjusted < 0.05; Supplementary Material 3, Table S6). This suggests that, overall, our data reflect ontogenetic trends rather than inter-annual variation in the perch population.

## Discussion

Feeding on the best quality resource is expected to be highly adaptive, likely playing an important role in life-history trade-offs of consumers. Our study shows that mismatches in the availability of physiologically important PUFAs, such as DHA, between a polymorphic aquatic consumer and its diet are associated with ontogenetic niche shifts and intra-cohort differences in growth. This partially agrees with other studies suggesting that nutritional quality of food may promote niche shifts (Vrede et al. 2011; Showalter et al. 2016). However, our results also suggest that the benefits of maximizing FA nutrition may also trade-off with other individual traits, such as local adaptations to specific habitats or specialization toward certain resources as well as the overall high ability of perch to regulate FA composition internally. If this is the case, perch may trade-off FA nutrition with classical selecting pressures in life history, such as risk of predation and food availability in different habitats. Overall, these results highlight the importance of FAs in life-history trade-offs in natural populations, strongly encouraging future efforts on studying the ecological and evolutionary implications of FAs across taxa.

### Dietary sources and internal regulation affect FA composition over ontogeny

In contrast to our expectations and the overall paradigm in FA ecology, in which changes in FA composition are mainly associated with diet (e.g. Napolitano 1999; Iverson 2009), diet explained a limited portion of the intraspecific FA variation in perch. Ontogenetic processes, represented by the wide range in perch total lengths, explained a similar proportion of FA variation (23%) as compared to diet (28%) during the early life of perch. Despite the previous evidence of ontogenetic FA changes associated with internal regulation (Lane et al. 2011; Maazouzi et al. 2011), this is the first quantitative and statistical support for such process over ontogeny of a consumer. Condition factor explained 1% of the remaining variation, suggesting a minor contribution of this fitness indicator on FA composition. Similarly, after subtracting the effect of diet, ontogeny and condition factor, habitat did not significantly affect FA composition suggesting either that adaptations or FA availability specific to habitat may not be an important factor affecting FA composition. Yet, 48% of the FA variation remained unexplained by the model, suggesting that other factors unrelated to the ones addressed in this study may also affect FA composition. Differences in FA composition may also depend on sex (Henderson et al. 1984), sexual maturity (Manor et al. 2012), temperature (Farkas et al. 1984), fasting (Abi-Ayad et al. 2000; Ballantyne et al. 2003; Taipale et al. 2011), pollutants (Janer et al. 2007), phenology (Blanchard et al. 2005; Rudchenko and Yablokov 2018; Keva et al. 2019), or possibly also genetic variation. Further studies including the above mentioned or other factors may therefore help to reveal the different drivers of FA composition and its potential ecological implications. All of these results together suggest that internal regulation may have stronger effects on FA composition over ontogeny of organisms than previously thought (Iverson 2009). This has major implications for how changes in FA composition are interpreted, from a view where the diet is the dominating factor, to a view where both external and internal processes are important.

### Dietary effects on FAs composition are related to trophic biomarkers

FA composition of perch was consistent with the availability of FA trophic biomarkers in the diet. As found in the previous studies, planktivorous perch had higher proportions of SDA and ALA (Scharnweber et al. 2016), which are more prevalent in planktonic prey (Lau et al. 2012; Scharnweber et al. 2016). Benthivorous perch were instead enriched in 18:1n-7, similar to most of the benthic macroinvertebrates that they feed on (Lau et al. 2012; Scharnweber et al. 2016). Piscivorous perch were mainly depleted in SDA and ALA, coinciding with extremely low proportions of SDA and ALA in fish as compared to invertebrates (Czesny et al. 2011; Lau et al. 2012). These results agree with the idea that certain FAs, especially those which do not carry out important physiological functions, are transferred to consumers with little modification (Iverson 2009) and therefore can be used as trophic biomarkers (Napolitano 1999; Iverson 2009). FA analysis is, therefore, a valuable tool to identify ontogenetic diet shifts or niche segregation in multiple taxa and environments (e.g. Iverson 2009; Ruess and Müller-Navarra 2019).

### Physiological shifts during life history are related to changes in FA composition

Ontogenetic patterns in single FAs did not always reflect dietary inputs, but rather FA regulation (or FA homeostasis) associated with changes in FA requirements over ontogeny. Proportions of EPA and ARA, which are highly bioactive FAs during sexual maturity and reproduction (Henderson and Tocher 1987), increased in perch dorsal muscle over early ontogeny and then decreased after perch reached 3+ years old, co-occurring with perch sexual maturation in the latitudes of this study (Heibo et al. 2005). This may indicate that perch accumulate EPA and ARA in muscle to be mobilized during sexual maturation, as suggested by the previous studies (Blanchard et al. 2005; Rudchenko and Yablokov 2018). In contrast, monounsaturated fatty acids (MUFAs), such as 16:1n-7 and 18:1n-9, which are important energy sources for fish (Tocher 2003), were found in low proportions in dorsal muscle over the early ontogeny of perch, while increasing monotonically after age 3+ irrespective to diet. Such ontogenetic increases of MUFAs have been described in multiple fish taxa, probably reflecting widespread life-history shifts in metabolism (e.g. Iverson et al. 2002; Maazouzi et al. 2011; Czesny et al. 2011). Juvenile fish likely catabolize MUFAs to maximize growth, thus avoiding predation and increasing size-specific fecundity (De Roos and Persson 2013), whereas adult individuals may compromise growth by storing energy-rich MUFAs in the tissues to be used over fasting periods or during reproduction (Tocher 2003).

DHA decreased over the ontogeny of the non-piscivorous perch, as found in multiple non-piscivorous fish (e.g. Ju et al. 1997; Iverson et al. 2002; Maazouzi et al. 2011) (but see exceptions in Jensen et al. 2007; Czesny et al. 2011). This suggests that most fish decrease DHA retention in muscle tissue over ontogeny, likely reflecting the higher requirements of DHA for growth, brain and eye development in early juveniles (Tocher 2010). However, such decreasing trends did not remain in adult piscivorous perch, probably due to higher DHA inputs from fish than invertebrates (Lau et al. 2012).

Despite DHA differences among perch prey (Lau et al. 2012; Scharnweber et al. 2016), DHA proportions in muscle did not differ substantially among perch with different diets. Benthivorous individuals experiencing low dietary DHA levels, may make up for this deficiency, either via (1) the selective retention of DHA, over other FAs (e.g. Abi-Ayad et al. 2000; Twining et al. 2016b) from minor contributions of DHA-rich zooplankton in diet and/or (2) the bioconversion of EPA and ALA found in benthic macroinvertebrates into DHA (Henderson and Tocher 1987; Tocher 2003). We found that DPA, an intermediate stage in EPA to DHA bioconversion (Bell and Tocher 2009; Iverson 2009), was more prevalent in the muscle of benthivorous than in planktivorous perch. Indeed, using compound-specific stable isotope analysis of EPA, ARA and DHA, Scharnweber et al. (unpublished manuscript) found evidence for bioconversion from EPA to DHA in benthivorous perch from this study. This may indicate that perch use EPA to DHA bioconversion to counteract low DHA availability in food. This agrees with Sawyer et al. (2016), who suggested that EPA to DHA bioconversion was the most important source of DHA in benthivorous yellow perch, *Perca flavescens* (Mitchil), using mass-balance modelling. However, the extrapolation of these results in perch to other consumer taxa may still be difficult as bioconversion abilities depend on the taxonomic identity of the consumer, being highest in invertebrates and lowest in terrestrial carnivorous vertebrates (Bell and Tocher 2009). Moreover, intraspecific bioconversion efficiencies may change over ontogeny (Henrotte et al. 2011). Therefore, additional studies are needed to determine to which extent PUFA bioconversion contributes to DHA inputs of consumers, and compensates for differences in DHA availability in dietary sources.

### The role of FAs in ontogenetic trade-offs

In this study, planktivorous pelagic perch had greater total length and weight as compared to planktivorous and benthivorous perch in the littoral zone during the first 2 years, which may indicate a gradual positive effect on growth by feeding on DHA-rich zooplankton in pelagic environments (Supplementary Material 3, Fig. S5). Such results are consistent with the higher availability of high-quality zooplankton prey in pelagic versus littoral environments (Ballantyne et al. 2003; Maazouzi et al. 2011), suggesting higher growth related to diets providing with DHA. However, high growth may be counteracted by higher predation risk in the open water, which is a major structuring force affecting the maintenance of littoral and pelagic resource polymorphism in perch (Eklöv and Svanbäck 2006; Svanbäck and Eklöv 2011). This study, like others, also found perch specializing in zooplankton in the littoral zone (Scharnweber et al. 2016; Bartels et al. 2016). Despite feeding on DHA-rich resources, these perch reached lower sizes at age than pelagic planktivorous perch and they did not have higher growth compared to perch feeding on low-DHA benthos. The morphological adaptations of littoral perch, which enhance manoeuvrability in complex habitats, often result in lower feeding efficiencies on planktonic than benthic prey (Svanbäck and Eklöv 2004; Scharnweber et al. 2016). Littoral perch specializing on zooplankton may deal with such morphological constraints, by consuming DHA-rich prey, while littoral benthivorous perch may instead rely on internal metabolic processes to obtain and retain DHA in tissues.

In this study, littoral perch shifted to piscivory at an earlier size and age than pelagic perch, which remained planktivorous at least until age 3+, agreeing with previous studies (Svanbäck and Eklöv 2002; Svanbäck et al. 2008). Observed differences in ontogenetic niche between littoral and pelagic zones may reflect differences in the availability of prey fish between the two environments. However, such a shift may also be advantageous for perch by allowing them to fulfil the high needs for DHA associated with reproduction (e.g. Tocher 2003; Henrotte et al. 2010), given the low availability of DHA in benthic diets (Lau et al. 2012; Scharnweber et al. 2016) and costs of feeding on planktonic diets in littoral habitats (Svanbäck and Eklöv 2004; Scharnweber et al. 2016). Indeed, piscivory was related to higher growth rates in 3+ perch, agreeing with the established idea that shifts to piscivory promote growth in fish (e.g. Mittelbach and Persson 1998; Litz et al. 2017). However, piscivory did not lead to higher growth rates in 1+ and 2+ perch. For those perch, lower foraging efficiency on larger fish prey at smaller sizes (Lundvall et al. 1999) may have counteracted the higher food quality and the energy content in prey fish (Mittelbach and Persson 1998; Lau et al. 2012).

By assessing fatty acids, we come to similar conclusions as Vrede et al. (2011), who found that resource stoichiometry would promote the shift to piscivory in perch. Despite the likelihood of long-chained PUFAs to limit consumer growth, we cannot disregard that other nutrients or biomolecules than FAs may also limit or co-limit growth and fitness at some point over ontogeny (e.g. Martin-Creuzburg et al. 2009; Ruess and Müller-Navarra 2019). Therefore, to test the mechanisms proposed in this correlative study, further experimental studies are needed, preferably including multiple potentially limiting nutrients in nature.

The current observational study draws its conclusions from biochemical signatures of an aquatic ectothermic consumer. However, recent results suggest that endothermic consumers may have similar or even higher requirements than fish for physiologically important long-chained PUFAs (Hulbert 2007; Twining et al. 2016b), while those FAs are much less prevalent at the base of terrestrial food webs (Hixson et al. 2015; Twining et al. 2016a; but see Ruess and Müller-Navarra 2019). Therefore, mismatches between food and consumer FA composition may also be an important driver of fitness and life-history trade-offs in endotherm organisms (Twining et al. 2016b, 2018), suggesting that the mechanisms portrayed in this study will likely be widespread in nature.

## Conclusion

Our study presents evidence that changes in internal regulation during ontogeny can have important effects in the FA composition of consumers that can be of a similar magnitude as the influence of diet. This has major implications for how to interpret the role of FAs in ecological studies, where within-species FA variation has been previously thought to mainly depend on diet (Iverson et al. 2002; Czesny et al. 2011). According to our results, internal FA regulation may buffer, to some extent, differences between the availability and physiological needs of FAs over ontogeny (e.g. Abi-Ayad et al. 2000; Taipale et al. 2011; Twining et al. 2016b). However, mismatches between needs for physiologically important FAs and the prevalence of those FAs in diet can affect both niche specialization and ontogenetic niche shifts in nature. We suggest that FA nutrition may be a common driver of life-history trade-offs in consumers, encouraging future efforts on studying the ecological and evolutionary implications of FA nutrition across taxa.

## Electronic supplementary material

Below is the link to the electronic supplementary material.

Supplementary material 1 (DOCX 297 kb)
