# Supplementary material for: Regulation of fatty acid composition related to ontogenetic changes and niche differentiation of a common aquatic consumer
Source: Oecologia. 2020 May 21;193(2):325–36. doi: 10.1007/s00442-020-04668-y (PMC7320933; doi:10.1007/s00442-020-04668-y)
Supplement: Supplementary file 1 — Supplementary material 1 (DOCX 297 kb) [file 442_2020_4668_MOESM1_ESM.docx]

**Supplementary material: Regulation of fatty acid composition related to ontogenetic changes and niche differentiation of a common aquatic consumer**

# F. Chaguaceda^*^ ^a^, P. Eklöv ^a^ and K. Scharnweber ^a^

a Department of Ecology and Genetics, Uppsala University, Uppsala, Sweden

* Corresponding author: [fernando.chaguaceda@ebc.uu.se](mailto:fernando.chaguaceda@ebc.uu.se)

# Supplementary material 1: Stable Isotope and Clustering Analysis

Bayesian hierarchical models

The use of Bayesian mixing models is a powerful tool for food-web and ecosystem studies in order to calculate the proportions of different sources in consumers diets and organic pools based on proportional acquisition of different elements in their diets (Parnell et al. 2013; Phillips et al. 2014; Stock and Semmens 2016a). The key features of Bayesian mixing models are the incorporation of uncertainty both in the input and in the outputs of the model, the possibility of using underdetermined models, which have more sources than the number of tracers plus one, and the use of previous information to correct for mathematically possible, yet less ecologically likely, dietary combinations (Moore and Semmens 2008; Hopkins and Ferguson 2012; Parnell et al. 2013). Some Bayesian mixing models also allow hierarchical designs, allowing for the introduction of factors and covariates which are likely to affect isotopic mixtures in the dataset (Semmens et al. 2009; Parnell et al. 2013; Stock and Semmens 2016b). However, to make sure that the results are ecologically relevant, the results of Bayesian mixing models should always

be compared with the raw isotopic data in order to assess whether the model reflects actual isotopic variation in resources and consumers (Phillips et al. 2014).

Model structure

In this study, a number of different mixing models were performed and compared with the actual raw isotope data depicted in a biplot. MixSIAR software version 3.1.10 was used (Stock and Semmens 2016a), which allowed hierarchical models that help to incorporate previous knowledge on how resources and consumer populations are structured (Semmens et al. 2009). Such knowledge is important in order to obtain better dietary estimations (Ward et al. 2010; Parnell et al. 2013; Phillips et al. 2014). The model chosen for further analysis was based on information about the structure of the consumer population and at the same time the one that, reflected more accurately isotopic variation in the dataset.

Perch populations in lakes often consist of two ecotypes, one in the pelagic zone feeding predominantly on zooplankton and one in the littoral zone mostly feeding on macroinvertebrates (Svanbäck and Eklöv 2003). However, recent results suggest that littoral perch have larger niche width than pelagic perch, with some individuals highly relying on planktonic pathways (Bartels et al. 2016; Marklund et al. 2019). Indeed, irrespective of the habitat where perch were caught, isotopic variation seemed to cluster in two groups, one relying on more benthic pathways and one relying more on planktonic pathways (Fig. S1). Such structure based on the preferred prey of perch populations, was tested using k-means clustering, which is a non-hierarchical method that requires a fixed k number of groups (Clarke et al. 2014). This method seeks to minimise within-group sums of squares about the k group centroids. This is equivalent to minimising some weighted combination of within-group resemblances between pairs of samples, as measures by a squared Euclidean distance coefficient (Clarke et al. 2014). To distinguish the perch individuals between one group relying on benthic diet and another one

relying on pelagic diet, the number of groups was fixed to k=2 (i.e. “planktonic pathway”,

“benthic pathway”) using δ13C and δ15N as variables. The resulting groups are shown in Fig. S1. Subsequently, the dataset was divided in perch predominantly relying on planktonic pathways and perch mostly relying on benthic pathways, which were analysed separately in the mixing model.

Perch populations are highly structured due to ontogenetic differences in size (Persson 1983; Persson et al. 2000). Therefore, ontogenetic structure was incorporated in the model by nesting the effect of perch individual within each age group, given that age and total length were positively correlated in the dataset. Total length was refrained from being used as a covariate because the model would calculate the effect of total length for each individual, which does not make sense biologically.

Consequently, perch individual and pathway cluster were included as fixed factors, with individual nested within benthic or planktonic pathway clusters in the MixSIAR model.

End-members, priors and error structure

Copepods, cladocerans, benthic macroinvertebrates and fish taxa were used as end-members in the model, correcting for isotopic trophic fractionation. Isotopic fractionation can vary on multiple environmental and internal drivers (e.g. McCutchan et al. 2003). Consequently, it is recommended to use fractionation factors determined experimentally with the same species and food mixtures (Gannes et al. 1997; Caut et al. 2008; Wolf et al. 2009). However, we lack direct experimental knowledge of the species at different ontogenetic stages and diets. Therefore, we chose to used robust and conservative estimations of fractionation factors of 0.4 ± 1.3 for δ13C and 3.4 ± 1.0 for δ15N based on Post (2002) that represent natural variation in trophic fractionation. Copepods and cladocerans were refrained from being pooled as pelagic zooplankton due to their distinct δ15N value (Figs S1 & S2). Informative priors used in other studies relied on dietary differences between littoral and pelagic perch (Scharnweber et al.

2016). However, those priors are not applicable in this study, due to littoral perch having a very broad niche width, with different individuals having planktivorous, benthivorous and piscivorous diets (Fig. S2, Fig. 1 in the manuscript). Therefore, a general prior distribution was used and the error structure used in the model was process error only, aiming for the estimation of single mixture points (Stock and Semmens 2016b).

In the model output, chain convergence was checked using Gelman-Rubin and Geweke diagnostics (Stock and Semmens 2016a) and the accuracy of the model was verified by inspecting the probability distribution of posteriors, which is also reflected in the standard deviation (SD) of the dietary estimations. Overall, the outputs followed the trends seen in the raw isotopic data (Fig. S2, Fig. 1 in Manuscript). The output of the model was summarized in mean ± SD proportions of the different resources in the biomass of each perch individual. Thereafter, the pelagic contribution to each perch was calculated by summing the contributions of copepods and cladocerans. Standard deviation estimates of planktivory was obtained by calculating the square roots of the sum of squares of the standard deviations of the contribution of copepods and cladocerans in perch diet.

Identifying niche groups of perch

For further comparison of variation in perch diet from different habitats, the individuals were clustered into different niche groups based on diet, obtained from stable isotope analysis, and habitat, measured as habitat when captured. Perch tend to have high habitat fidelity (Eklöv 1997), therefore we assumed that perch habitat use where captured was a meaningful measure of their habitat use over the long term. Grouping to dietary niche was achieved with k-means clustering, based on individual dietary proportions of zooplankton, macroinvertebrates and fish in the diet obtained from Mix-SIAR models. The number of groups was fixed to k=3, aiming to obtain the three main ontogenetic diet shifts in perch (i.e. planktivorous, benthivorous and piscivorous perch). The validity of these diet groups was visually assessed in the isospace biplot

(Fig. S2). The groups found were littoral benthivorous (LB), littoral planktivorous (LP), pelagic planktivorous (PP), pelagic benthivorous (PB) and littoral piscivorous (Pisc). As only two perch were PB, this group was disregarded for further statistical tests.


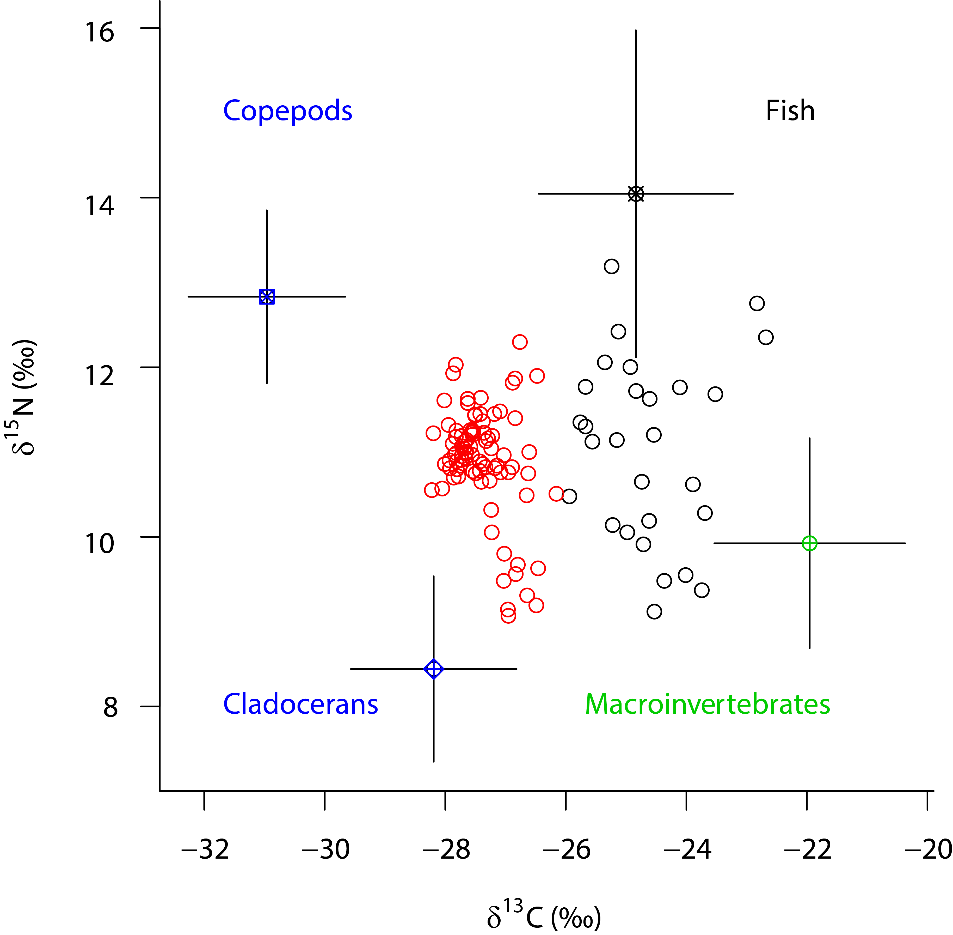


Figure S1. Isospace plot showing δ13C and δ15N values of perch from different pathways. Red symbols represent perch relying on the planktonic pathway, while black symbols represent perch relying on the benthic pathway. Crossed symbols and error bars represent the mean ± SD values of the main food sources of perch after correction of trophic fractionation.


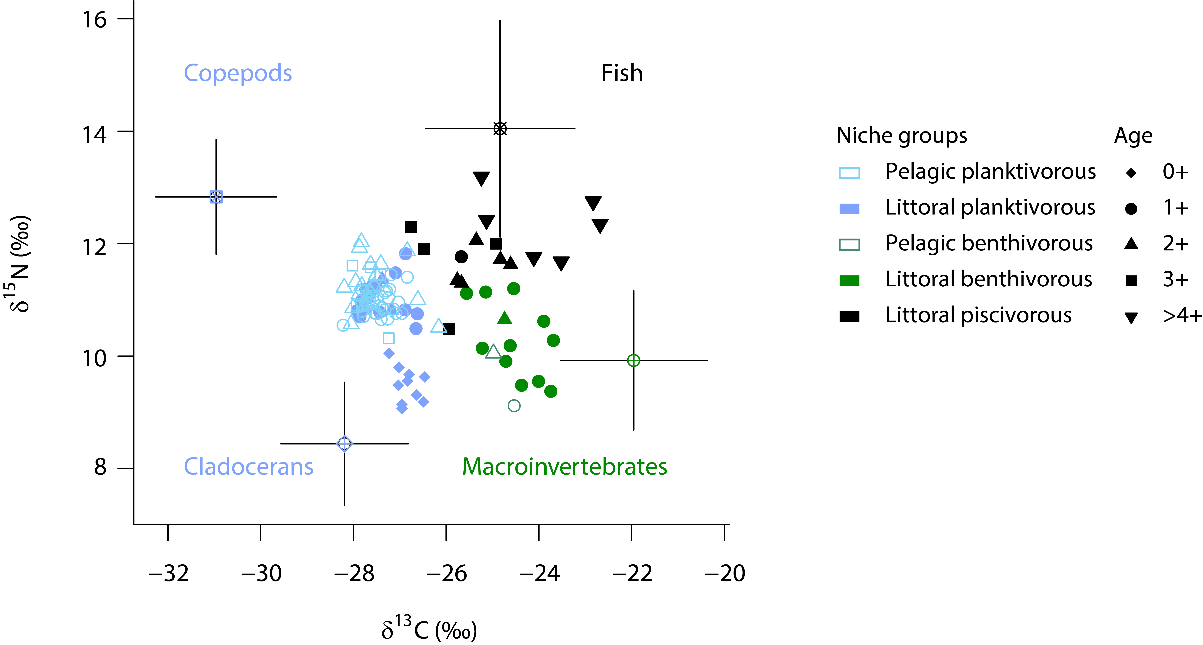


Figure S2. Isospace plot of perch based on δ13C and δ15N values of different niche groups of perch. Crossed symbols and error bars represent the mean ± SD values of the main food sources of perch after correction of trophic fractionation.

# Supplementary material 2: Fatty Acids

Fatty acid analyses

A piece of muscle tissue of perch was submerged in a solution of chloroform/methanol (2/1 by volume), thoroughly squeezed with a glass rod and sonicated for 10 minutes in iced water to enhance lipid to dissolve. An aqueous solution of 0.88 % potassium chloride was added followed by vortexing and centrifugation to remove non-lipids. The lipid extraction was repeated; then the two organic phases were pooled and evaporated under nitrogen stream. Then, lipid extracts were dissolved in hexane and transmethylated at 90°C for 90 minutes using 1% H2SO4 in methanol as a catalyst.

FA methyl esters (FAMEs) were analysed using an Agilent 6890 N Gas Chromatographer (Agilent Technologies, Santa Clara, 181 CA, USA) equipped with a DB-23 column (length 30 m, ID 0.25 mm, film thickness 0.25 μm; Agilent) at Uppsala University.

The samples were introduced with a 20:1 split injection at an initial temperature of 180°C for 8min, then increasing by 2°C min-1 until reaching 210°C, maintaining this temperature for 2 min. Helium was used as a carrier gas with an average velocity of 34.09 cm sec-1.

Mass spectrometry

In the chromatogram, the different FA peaks were identified using the NIST mass spectral search program (version 2.2) for the NIST/EPA/NIH Mass Spectral Library (NIST v14) (National Institute of Standards and Technology, U.S. Department of Commerce), retention times, and mass spectra (Christie 2018). Heneicosanoic acid (Nu-Chek, Prep, Elysian, Minnesota, USA) was used as internal standard. The areas under each peak were measured manually using MSD ChemStation F.01.01.2317 (Agilent Technologies, Inc.).

Calibration curve

Peak areas of the gas chromatography (GC) measurements were calibrated by running known FAME mixtures (GLC Reference standard 68 D; Nu-Chek Prep) at concentrations of a similar range found on the samples (0.2, 0.5, 1, 2, 5, 7, 10 µg µL-1) and with the same GC method. The calibration coefficient was calculated for each set of samples by fitting a linear regression between peak areas and known quantities of the different FAMEs. The calibration coefficient applied to each FA was the one of the known mixture FA of closest proximity. FA concentrations were also calculated based on the wet weight of the tissue samples in order to help interpret changes in proportions of FAs over the ontogeny of perch.

Supplementary results of FA analysis

To detect FA differences among diet and age groups, Similarity percentages routine (SIMPER) was used. To minimise the strength of relationship between diet and ontogeny a two-way crossed procedure was used, which summarizes dissimilarities within diet blocks and age blocks respectively (Clarke et al. 2014). As SIMPER routine does not provide a *P* -value, multivariate analysis was used to test for significances of the comparisons (Clarke et al. 2014). Age-0+ perch had significantly different FA composition compared to age-1+ from any of the feeding groups (pairwise PERMANOVA, *t*>2.61, *P* <0.001). Age 1+–3+ perch showed significant trends due to diet and ontogeny according to DistLM analysis shown in the manuscript, and >3+ perch had a significantly different FA composition than 3+ perch from any of the feeding groups (pairwise PERMANOVA, *t*>2.26, *P* <0.05). Results for FAs related to diet are shown in Table S1, while the ones related to ontogenetic changes are shown in Table S2. Equations of trends of single FAs related to ontogeny are shown in Table S3

Table S1. Differences in the FA composition of perch (*Perca fluviatilis* L.) with different diets.

a) Relative contribution of FA indicators to all FA dissimilarities between benthivorous and planktivorous perch, and non-piscivorous and piscivorous perch calculated using two-way crossed SIMPER analysis including age group as a cofactor. The foraging group with the highest content of each FA is denoted with letters (p, plankthivorous; b, benthivorous; nP, non- piscivorous; P, piscivorous). b) Mean contribution of each FA to the overall FA dissimilarity due to diet, based on the mean of the relative contributions for all pairwise diet comparisons. c) Cumulative contributions of FA indicators to FA dissimilarity. Only the five FAs having the highest mean contribution to FA dissimilarity are shown. LB, littoral benthivorous; LP, littoral planktivorous; PB, pelagic benthivorous; PP, pelagic planktivorous; Pisc, littoral piscivorous perch.

|  | a) Contribution to FA dissimilarity in each pairwise diet comparison (%) | | b) Mean contribution  to FA dissimilarity (%) | c) Cumulative contribution to FA dissimilarity (%) |
| --- | --- | --- | --- | --- |
|  | Benthivorous (LB+PB)  –  Planktivorous perch (LP+PP) | Non piscivorous (LP+PP+LB+PB)  –  Piscivorous perch (Pisc) |  |  |
| 18:4n-3 (SDA) | 14.7 p | 19.4 nP | 17.1 | 17.1 |
| 22:6n-3 (DHA) | 6.0 b | 13.0 P | 9.4 | 26.5 |
| 18:1n-7 | 8.0 b | 7.2 P | 7.6 | 34.1 |
| 18:3n-3 (ALA) | 6.3 p | 8.8 nP | 7.6 | 41.7 |
| 22:5n-3 (DPA) | 11.3 b | 4.0 P | 7.4 | 49.1 |

Table S2. Ontogenetic differences in the FA composition of perch. a) Relative contribution of FA indicators to all FA dissimilarities for each year-to-year comparison calculated using two- way crossed SIMPER analysis with diet group of perch as cofactor. b) Mean contribution of each FA to the overall FA dissimilarity over ontogeny, based on the mean of the relative contributions for all year-to-year comparisons. c) Cumulative contributions of FA indicators to FA dissimilarity. Only the five FAs having the highest mean contribution to FA dissimilarity are shown.

|  | Contribution to FA dissimilarity in year-to-year comparisons (%) | | | | | | b) Mean contribution  to FA  dissimilarity (%) | c) Cumulative contribution to  FA  dissimilarity (%) |
| --- | --- | --- | --- | --- | --- | --- | --- | --- |
| FA | 0+–1+ | 1+–2+ | 2+–3+ | 3+–4+ | 4+–5+ | 5+–6+ |  |  |
| 22:6n-3 (DHA) | 18.0 | 15.3 | 20.7 | 26.4 | 40.9 | 32.7 | 25.7 | 25.7 |
| 16:1n-7 | 5.6 | 3.6 | 6.8 | 18.3 | 7.4 | 19.1 | 11.4 | 35.8 |
| 20:5n-3 (EPA) | 2.2 | 7.3 | 8.4 | 10.3 | 10.9 | 5.3 | 7.4 | 43.2 |
| 18:1n-9 | 2.1 | 3.4 | 2.5 | 13.4 | 9.1 | 4.1 | 5.8 | 49.0 |
| 20:4n-6 (ARA) | 4.2 | 17.1 | 4.2 | 4.2 | 1.8 | 1.9 | 5.6 | 54.5 |

Table S3. Regressions of the five most responsive fatty acids (FAs) over ontogeny for the different niche groups of perch (PP, pelagic planktivorous; LP, littoral planktivorous; LB, littoral benthivorous and Pisc, littoral piscivorous.) using perch total length (*L*T) and *L*T 2 as independent variables. In the absence of significance of a quadratic model, linear models are shown. Goodness of fit of the model is evaluated with the adjusted R2 (Adj. R2). SE represents Standard Error of each estimate. Significant models are shown in bold.

| **FA (%)** | **Niche group** | **Estimation ± SE** | | | ***P*** | | | **Adj. R2** |
| --- | --- | --- | --- | --- | --- | --- | --- | --- |
|  |  | ***L*T 2**  (×10^-5^) | ***L*_T_**  (×10^-3^) | **intercept** | ***L*T 2** | ***L*_T_** | **intercept** |  |
| **16:1n-7** | PP |  | **-10±2** | **3.65±0.20** |  | **<0.001** | **<0.001** | **0.35** |
|  | LP |  | **-8±3** | **3.44±0.26** |  | **0.007** | **<0.001** | **0.22** |
|  | LB |  | -6±3 | 3.39±0.27 |  | 0.072 | <0.001 | 0.11 |
|  | Pisc |  | **26±6** | **-0.91±0.97** |  | **<0.001** | **0.365** | **0.59** |
| **18:1n-9** | PP | **-19.6±6.0** | **36±12** | **3.62±0.60** | **0.002** | **0.005** | **<0.001** | **0.17** |
|  | LP |  | 4±3 | 4.71±0.30 |  | 0.210 | <0.001 | 0.02 |
|  | LB |  | **-17±4** | **5.98±0.32** |  | **<0.001** | **<0.001** | **0.48** |
|  | Pisc |  | **31±5** | **0.17±0.89** |  | **<0.001** | **0.853** | **0.71** |
| **20:4n-6 (ARA)** | PP | **56.4±8.8** | **-72±18** | **8.57±0.87** | **<0.001** | **<0.001** | **<0.001** | **0.82** |
|  | LP |  | **13±4** | **5.61±0.37** |  | **0.003** | **<0.001** | **0.27** |
|  | LB |  | 1±5 | 6.28±0.43 |  | 0.821 | <0.001 | -0.05 |
|  | Pisc | **-39.0±18.0** | **139±65** | **-3.01±5.63** | **0.049** | **0.053** | **0.602** | **0.16** |
| **20:5n-3 (EPA)** | PP |  | **29±4** | **11.46±0.46** |  | **<0.001** | **<0.001** | **0.46** |
|  | LP | **97.5±32.0** | **-151±54** | **18.76±2.10** | **0.005** | **0.010** | **<0.001** | **0.34** |
|  | LB |  | **51±10** | **10.42±0.88** |  | **<0.001** | **<0.001** | **0.53** |
|  | Pisc |  | **-48±10** | **21.34±1.68** |  | **<0.001** | **<0.001** | **0.61** |
| **22:6n-3 (DHA)** | PP |  | **-35±9** | **24.71±1.01** |  | **<0.001** | **<0.001** | **0.19** |
|  | LP | **-272±110** | **451±185** | **5.58±7.21** | **0.020** | **0.022** | **0.446** | **0.14** |
|  | LB |  | -4±19 | 22.57±1.63 |  | 0.822 | <0.001 | -0.05 |
|  | Pisc |  | 114±197 | 12.92±16.97 |  | 0.575 | 0.460 | -0.12 |

To test for the effect of habitat on the FA composition of perch, we performed a PERMANOVA analysis on non-piscivorous perch, because piscivorous perch were only found in the littoral zone. Habitat had a significant effect on FA composition (Table S4a). However, once removing the FA variation explained by diet, total length and body condition (used for the DistLM analysis), habitat was not a significant factor affecting FA composition in perch (Table S4b).

A graphical comparison of the FA composition of the different perch resources is shown in Fig. S3. The general trends for total FA concentration are shown in Fig. S4 to facilitate interpretation of the changes of FA proportions in the manuscript.

Table S4. PERMANOVA models to identify the effect of habitat on the FA composition of non-piscivorous perch. a) Under a reduced model, habitat had a significant effect on FA composition. b) Model including covariates used in DistLM analysis and P obtained after 9999 Monte Carlo permutations.

|  | Source | d.f. | Sum of squares | Mean squares | Pseudo-*F* | *P* |
| --- | --- | --- | --- | --- | --- | --- |
| a) One-way  model | Habitat | 1 | 0.045 | 0.045 | 7.536 | < 0.001 |
|  | Residuals | 95 | 0.572 | 0.006 |  |  |
|  | Total | 96 | 0.617 |  |  |  |
| b) One-way  model with covariates | % planktivory | 1 | 0.141 | 0.141 | 44.129 | < 0.001 |
|  | *  *L*T | 1 | 0.141 | 0.141 | 43.970 | < 0.001 |
|  | % piscivory | 1 | 0.029 | 0.029 | 9.204 | < 0.001 |
|  | Body condition | 1 | 0.007 | 0.007 | 2.347 | 0.036 |
|  | Habitat | 1 | 0.006 | 0.006 | 1.875 | 0.078 |
|  | Residuals | 91 | 0.292 | 0.003 |  |  |
|  | Total | 96 | 0.618 |  |  |  |

* Total length


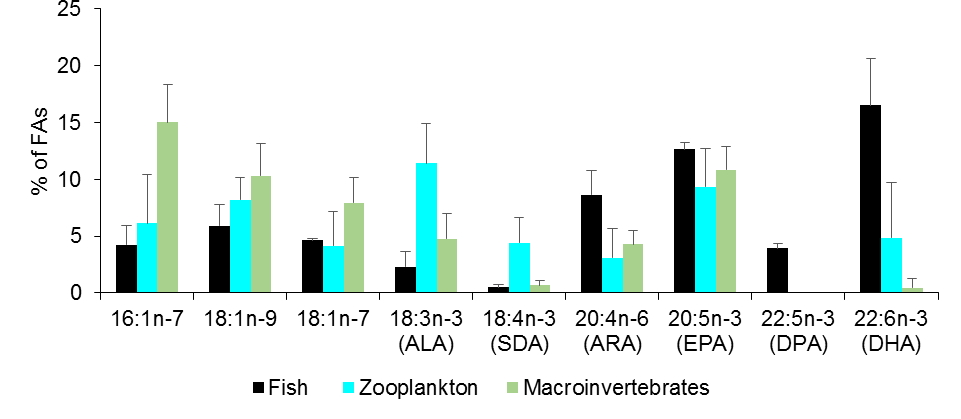


Figure S3. Fatty acid proportions of the different food sources of perch (mean ± SD). Fish FA proportions are calculated from the mean of roach (*Rutilus rutilus* L*.*) and ruffe (*Gymnocephalus cernua* L.), which were the main prey fish found in Lake Erken during the time of the study. Zooplankton and macroinvertebrate FA values represent the mean of different taxa from Scharnweber et al. (2016).


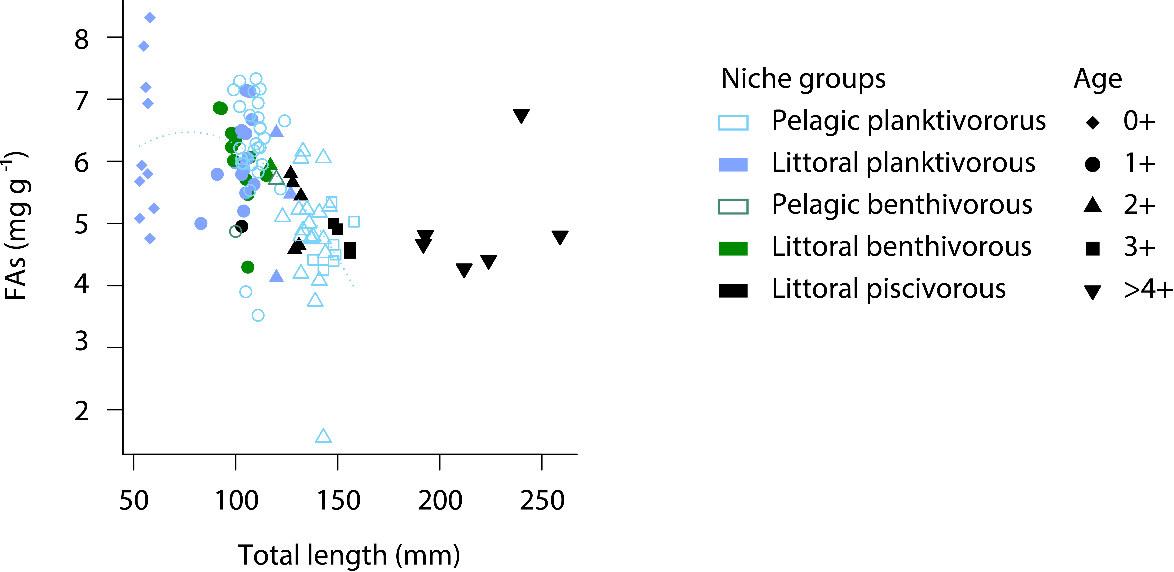


Figure S4. Ontogenetic trends of the concentrations of FAs in the dorsal muscle of perch in relation to total perch length for the different niche groups of perch (see legend). Dashed cyan lines represent significant linear models for pelagic planktivorous perch.

**Supplementary material 3: Growth and Condition Analysis**

Non-parametric statistics equivalent to ANOVA designs and post hoc comparisons, were used in order to test the differences in total length, weight and condition factor of perch at the time of catch (Table S5). The same statistics were used to test intra-cohort differences in growth among the different niche groups of perch (PP, pelagic planktivorous; PB, pelagic benthivorous; LP, littoral planktivorous; LB, littoral benthivorous; Pisc, littoral piscivorous) and inter-cohort differences within each niche group of perch (Table S6; Fig. S5). The groups defined for these tests were based on cohort and niche group. For example, LP 1 means littoral planktivorous perch that hatched one year before the study (i.e. 2014), therefore belonging to cohort 2014.

The reason for using non-parametric tests was the robustness of those tests to non-normal distribution of residuals assuming that the variances are homogenous across groups, which was the case. Kruskal-Wallis rank sum test was used in order to see if the mean of any of the groups were significantly different while Dunn test on multiple comparison with Benjamini-Hochberg correction, (Ogle 2017) was used for post-hoc pairwise comparisons between groups. Groups, with only one individual were excluded and thus, Pisc 1, LB 2, PB 1 and PB 2 perch were excluded from the analysis. Non-parametric tests were calculated using R version 3.6.1 (R Core Team 2019).

**Supplementary material 4: Data Summary**

A summary of the different variables for each age and niche group is presented in Table S7.

Table S5. Non-parametric tests comparing means in total length (*L*T), weight and condition factor for the different groups of perch based on cohort and niche group. a) Kruskal-Wallis rank sum test for the different age groups or cohorts of perch. b) Dunn test on multiple comparison with Benjamini-Hochberg correction to test for pairwise differences between the groups based on cohort and niche group. Significant results are highlighted in bold font. LB, littoral benthivorous; LP, littoral planktivorous; PB, pelagic benthivorous; PP, pelagic planktivorous; Pisc, littoral piscivorous perch.

|  | a) Kruskal-Wallis test | | | b) Dunn test | | |
| --- | --- | --- | --- | --- | --- | --- |
| Variable | Age group /  Cohort | χ2 (d.f.) | *P* | Comparison | *Z* | *P-*adj. |
| *L*T | **Age 1+ /**  **Cohort 2014** | **14.08**  **(2)** | **<0.01** | LB 1 - LP 1 | -0.32 | 0.75 |
|  |  |  |  | **LB 1 - PP 1** | **-3.07** | **0.01** |
|  |  |  |  | **LP 1 - PP 1** | **-3.01** | **<0.01** |
|  | **Age 2+ /**  **Cohort 2013** | **13.81**  **(2)** | **<0.01** | LP 2 - Pisc 2 | -0.83 | 0.40 |
|  |  |  |  | **LP 2 - PP 2** | **-3.06** | **0.01** |
|  |  |  |  | **Pisc 2 - PP 2** | **-2.57** | **0.02** |
|  |  |  |  | **LP 2 - PP 2** | **-3.06** | **0.01** |
|  | Age 3+ /  Cohort 2012 | 2.34 (1) | 0.13 |  | | |

Table S5. Continued.

|  | a) Kruskal-Wallis test | | | b) Dunn test | | |
| --- | --- | --- | --- | --- | --- | --- |
| Variable | Age group /  Cohort | χ2 (d.f.) | *P* | Comparison | Z | *P*-adj. |
| Weight | **Age 1+ /**  **Cohort 2014** | **21.39**  **(2)** | **< 0.01** | LB 1 - LP 1 | -0.16 | 0.87 |
|  |  |  |  | **LB 1 - PP 1** | **-3.64** | **< 0.01** |
|  |  |  |  | **LP 1 - PP 1** | **-3.85** | **< 0.01** |
|  | **Age 2+ /**  **Cohort 2013** | **16.61**  **(2)** | **< 0.01** | LP 2 - Pisc 2 | -0.51 | 0.61 |
|  |  |  |  | **LP 2 - PP 2** | **-3.10** | **<0.01** |
|  |  |  |  | **Pisc 2 - PP 2** | **-3.11** | **0.01** |
|  |  |  |  | **LP 2 - PP 2** | **-3.10** | **<0.01** |
|  | Age 3+ /  Cohort 2012 | 3.57 (1) | 0.06 |  | | |
| Condition factor | **Age 1+ /**  **Cohort 2014** | **6.01 (2)** | **0.049** | LB 1 - LP 1 | -0.22 | 0.82 |
|  |  |  |  | LB 1 - PP 1 | -2.01 | 0.13 |
|  |  |  |  | LP 1 - PP 1 | -1.96 | 0.07 |
|  | Age 2+ /  Cohort 2013 | 5.24 (2) | 0.07 |  | | |
|  | Age 3+ /  Cohort 2012 | 2.29 (1) | 0.13 |  |  |  |

Table S6. Non-parametric tests on the growth between consecutive years and length-at-age for the different groups of perch. a) Kruskal-Wallis rank sum test for different ages of perch. b) Dunn test on multiple comparison with Benjamini-Hochberg correction to test pairwise differences between the groups within and among cohorts. Significant results are highlighted in bold font. LB, littoral benthivorous; LP, littoral planktivorous; PB, pelagic benthivorous; PP, pelagic planktivorous; Pisc, littoral piscivorous perch.

|  | a) Kruskall-Wallis  test | | | b) Dunn test | | | | |
| --- | --- | --- | --- | --- | --- | --- | --- | --- |
| Variable | Age (year) | χ2 (d.f.) | *P* | Type of comparison | Group | Comparison | Z | *P*-adj. |
| Length- at-age | **1** | **41.81**  **(7)** | **< 0.01** | Intra-cohort | Cohort 2014 | LB 1–LP 1 | -1.39 | 0.38 |
|  |  |  |  |  |  | **LB 1–PP 1** | **-3.87** | **<0.01** |
|  |  |  |  |  |  | **LP 1–PP 1** | **-2.58** | **0.04** |
|  |  |  |  |  | Cohort 2013 | LP 2–Pisc 2 | -0.18 | 0.89 |
|  |  |  |  |  |  | LP 2–PP 2 | -1.11 | 0.44 |
|  |  |  |  |  |  | Pisc 2–PP 2 | -1.11 | 0.42 |
|  |  |  |  |  | Cohort  2012 | Pisc 3–PP 3 | -1.24 | 0.43 |
|  |  |  |  | Inter-cohort | LP | LP 1–LP 2 | 1.71 | 0.24 |
|  |  |  |  |  | PP | **PP 1–PP 2** | **4.18** | **<0.01** |
|  |  |  |  |  |  | **PP 1–PP 3** | **3.21** | **0.01** |
|  |  |  |  |  |  | PP 2–PP 3 | 0.31 | 0.85 |
|  |  |  |  |  | Pisc | Pisc 2–Pisc 3 | 0.53 | 0.73 |
|  | **2** | **17.5 (4)** | **< 0.01** | Intra-cohort | Cohort 2013 | LP 2–Pisc 2 | 0.03 | 0.97 |
|  |  |  |  |  |  | **LP 2–PP 2** | **-2.60** | **0.046** |
|  |  |  |  |  |  | **Pisc 2–PP 2** | **-3.27** | **0.01** |
|  |  |  |  |  | Cohort 2012 | Pisc 3–PP 3 | -1.39 | 0.27 |
|  |  |  |  | Inter-cohort | Pisc | Pisc 2–Pisc  3 | -0.57 | 0.71 |
|  |  |  |  |  | PP | PP 2–PP 3 | 0.87 | 0.55 |
|  | 3 | 3.57 (1) | 0.06 |  | | | | |

Table S6. Continuation

|  | a) Kruskall-Wallis  test | | | b) Dunn test | | | | |
| --- | --- | --- | --- | --- | --- | --- | --- | --- |
| Variable | Age (year) | χ2 (d.f.) | *P* | Type of comparison | Group | Comparison | Z | *P*-adj. |
| Growth | **1–2** | **9.80 (4)** | **0.04** | Intra-cohort | Cohort 2013 | LP 2–Pisc 2 | 0.08 | 0.94 |
|  |  |  |  |  |  | LP 2–PP 2 | -2.00 | 0.23 |
|  |  |  |  |  |  | Pisc 2–PP 2 | -2.60 | 0.09 |
|  |  |  |  |  | Cohort 2012 | Pisc 3–PP 3 | -0.41 | 0.76 |
|  |  |  |  | Inter-cohort | PP | PP 2–PP 3 | 1.07 | 0.47 |
|  |  |  |  |  | Pisc | Pisc 2–Pisc 3 | -0.85 | 0.56 |
|  | **2–3** | **6.04 (1)** | **0.01** | Intra-cohort | Cohort 2012 | **Pisc 3–PP3** | – | – |


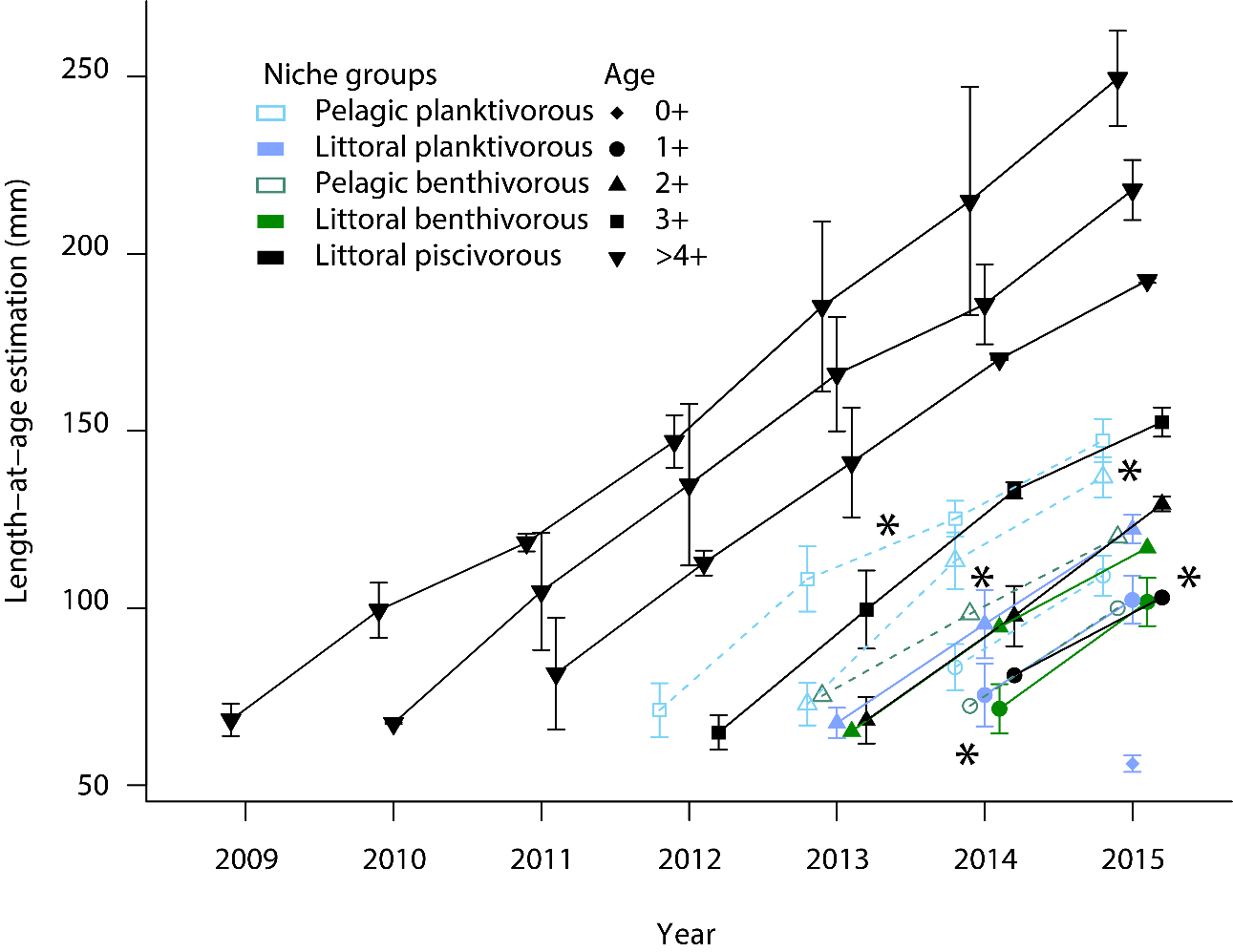


Figure S5. Length-at-age estimations of the different niche groups of perch (see legend). The lines represent mean individual growth trajectories of different cohorts from different niche groups: dashed cyan lines represent pelagic planktivorous perch, dashed blue-green lines represent pelagic benthivorous green solid lines represent littoral benthivorous, dark blue solid lines represent littoral planktivorous and black solid lines littoral piscivorous perch. Asterisks mean significant differences (*P*<0.05) of length-at-age and growth in intra-cohort comparisons of different niche groups of perch.

Table S7. Means ± standard deviation (SD) of the main variables of perch (*Perca fluviatilis* L.) from different niche groups (LP, littoral planktivorous; LB, littorral benthivorous; PP, pelagic planktivorous; PB, pelagic benthivorous; Pisc, littoral piscivorous) and of different age (1–6). Combinations of both classifiers are shown as niche group followed by age. Fatty acids in bold represent the most responsive to changes either in diet or in ontogeny of perch according to SIMPER analysis (see Supplementary material 2 for more information).

|  | **Planktivorous in the littoral zone** | | | **Benthivorous in the littoral zone** | | **Planktivorous in the pelagic zone** | | | **Benthivorous in the pelagic zone** | |  |  | **Piscivorous** | |  |  |
| --- | --- | --- | --- | --- | --- | --- | --- | --- | --- | --- | --- | --- | --- | --- | --- | --- |
| **LP 0** | | **LP 1** | **LP 2** | **LB 1** | **LB 2** | **PP 1** | **PP 2** | **PP 3** | **PB 1** | **PB 2** | **Pisc 1** | **Pisc 2** | **Pisc 3** | **Pisc 4** | **Pisc 5** | **Pisc 6** |
| *n* a | 10 | 15 | 3 | 11 | 1 | 28 | 20 | 7 | 1 | 1 | 1 | 5 | 4 | 2 | 2 | 2 |
| Weight (g) | 1.6 ± 0.2 | 11.5 ± 2.2 | 18.3 ± 1.2 | 11.3 ± 2.3 | 19.4 | 14.7 ± 2.1 | 27.1 ± 3.3 | 32.0 ± 5.7 | 12.2 | 21.9 | 11.9 | 20.7 ± 1.6 | 39.0 ± 3.7 | 93.5 ± 22.0 | 118.5 ± 17.1 | 210.1 ± 52.4 |
| *L* _T_ b (mm) | 56.1 ± 2.3 | 102.3 ± 6.8 | 122.3 ± 4.0 | 101.7 ± 6.8 | 117 | 109.1 ± 5.6 | 136.9 ± 5.7 | 147.3 ± 6.1 | 100 | 120 | 103 | 129.4 ± 2.1 | 152.5 ± 4.1 | 192.5 ± 0.7 | 218.0 ± 8.5 | 249.5 ± 13.4 |
| Condition | 0.9 ± 0.1 | 1.1 ± 0.1 | 1.0 ± 0.1 | 1.1 ± 0.1 | 1.2 | 1.1 ± 0.1 | 1.1 ± 0.1 | 1.0 ± 0.1 | 1.2 | 1.3 | 1.1 | 1.0 ± 0.1 | 1.1 ± 0.0 | 1.3 ± 0.3 | 1.1 ± 0.0 | 1.3 ± 0.1 |
| Length-at-age 1 (mm) | - | 75.5 ± 8.8 | 67.6 ± 4.3 | 71.6 ± 7.0 | 65.2 | 83.4 ± 6.5 | 72.9 ± 6.0 | 71.2 ± 7.6 | 72.4 | 75.2 | 81 | 68.3 ± 6.6 | 64.9 ± 4.8 | 81.5 ± 15.8 | 67.5 ± 0.2 | 68.4 ± 4.6 |
| Length-at-age 2 (mm) | - | - | 95.5 ± 9.6 | - | 94.7 | - | 113.3 ± 8.0 | 108.2 ± 9.2 | - | 98.4 | - | 97.8 ± 8.5 | 99.6 ± 11.0 | 112.7 ± 3.5 | 104.7 ± 16.6 | 99.4 ± 7.7 |
| Length-at-age 3 (mm) | - | - | - | - | - | - | - | 125.3 ± 5.0 | - | - | - | - | 133.2 ± 2.3 | 141.1 ± 15.5 | 134.8 ± 22.8 | 118.5 ± 2.5 |
| Length-at-age 4 (mm) | - | - | - | - | - | - | - | - | - | - | - | - | - | 170.3 ± 0.3 | 166.0 ± 16.1 | 147.1 ± 7.4 |
| Length-at-age 5 (mm) | - | - | - | - | - | - | - | - | - | - | - | - | - | - | 185.7 ± 11.3 | 185.1 ± 24.0 |
| Length-at-age 6 (mm) | - | - | - | - | - | - | - | - | - | - | - | - | - | - | - | 214.9 ± 32.2 |
| δ^13^C (‰) | -26.8 ± 0.2 | -27.4 ± 0.5 | -27.4 ± 0.3 | -24.5 ± 0.6 | -24.7 | -27.4 ± 0.3 | -27.6 ± 0.5 | -27.7 ± 0.3 | -24.5 | -25 | -25.7 | -25.2 ± 0.5 | -26.0 ± 0.8 | -24.3 ± 1.1 | -24.7 ± 0.8 | -22.8 ± 0.1 |
| δ^15^N (‰) | 9.5 ± 0.3 | 11.0 ± 0.3 | 11.0 ± 0.3 | 10.3 ± 0.7 | 10.7 | 10.9 ± 0.2 | 11.3 ± 0.4 | 11.1 ± 0.5 | 9.1 | 10.1 | 11.8 | 11.6 ± 0.3 | 11.7 ± 0.8 | 12.1 ± 0.5 | 12.5 ± 1.0 | 12.6 ± 0.3 |
| Planktivory (%) | 73.7 ± 2.1 | 65.4 ± 4.8 | 66.5 ± 3.6 | 25.0 ± 4.0 | 24 | 67.8 ± 3.8 | 66.4 ± 5.1 | 70.0 ± 2.7 | 27.2 | 27.5 | 28.7 | 24.6 ± 4.3 | 36.1 ± 10.9 | 15.5 ± 4.0 | 15.5 ± 1.3 | 7.6 ± 0.1 |
| Benthivory (%) | 17.7 ± 1.6 | 12.1 ± 1.8 | 11.7 ± 1.2 | 55.8 ± 7.8 | 46.6 | 13.4 ± 1.5 | 12.7 ± 3.1 | 12.2 ± 1.1 | 60.3 | 46.6 | 37.5 | 35.5 ± 3.5 | 23.5 ± 12.1 | 37.0 ± 11.1 | 28.2 ± 11.0 | 43.1 ± 3.3 |
| Piscivory (%) | 8.6 ± 1.0 | 22.5 ± 3.5 | 21.8 ± 3.0 | 19.2 ± 5.4 | 29.4 | 18.8 ± 2.5 | 20.9 ± 3.5 | 17.8 ± 2.3 | 12.5 | 25.9 | 33.8 | 39.8 ± 3.2 | 40.3 ± 5.7 | 47.5 ± 7.0 | 56.1 ± 9.7 | 49.2 ± 3.5 |
| 14:0 (%) | 1.0 ± 0.2 | 1.0 ± 0.2 | 1.3 ± 0.2 | 0.8 ± 0.2 | 0.5 | 1.2 ± 0.1 | 1.0 ± 0.1 | 0.9 ± 0.2 | 0.8 | 0.9 | 0.9 | 0.7 ± 0.0 | 0.5 ± 0.0 | 0.7 ± 0.3 | 0.8 ± 0.2 | 0.8 ± 0.2 |
| i-15:0 (%) | 0.6 ± 0.2 | 0.4 ± 0.1 | 0.5 ± 0.1 | 0.3 ± 0.1 | 0.1 | 0.4 ± 0.1 | 0.3 ± 0.1 | 0.2 ± 0.1 | 0.4 | 0.2 | 0.2 | 0.2 ± 0.0 | 0.1 ± 0.0 | 0.1 ± 0.0 | 0.1 ± 0.0 | 0.1 ± 0.1 |
| ai-15:0 (%) | 0.3 ± 0.1 | 0.2 ± 0.1 | 0.3 ± 0.1 | 0.1 ± 0.0 | 0 | 0.2 ± 0.0 | 0.1 ± 0.0 | 0.1 ± 0.0 | 0.1 | 0.1 | 0.1 | 0.0 ± 0.0 | 0.0 ± 0.0 | 0.0 ± 0.0 | 0.0 ± 0.0 | 0.0 ± 0.0 |
| 15:0 (%) | 0.4 ± 0.0 | 0.4 ± 0.0 | 0.4 ± 0.0 | 0.4 ± 0.0 | 0.4 | 0.4 ± 0.0 | 0.4 ± 0.0 | 0.4 ± 0.0 | 0.5 | 0.3 | 0.3 | 0.4 ± 0.0 | 0.3 ± 0.0 | 0.3 ± 0.0 | 0.3 ± 0.0 | 0.3 ± 0.1 |
| i-16:0 (%) | 0.3 ± 0.1 | 0.3 ± 0.1 | 0.4 ± 0.0 | 0.5 ± 0.3 | 0.4 | 0.3 ± 0.1 | 0.4 ± 0.1 | 0.4 ± 0.1 | 1.1 | 0.6 | 0.4 | 0.4 ± 0.1 | 0.8 ± 0.4 | 0.6 ± 0.0 | 0.6 ± 0.3 | 0.4 ± 0.0 |
| ai-16:0 (%) | 0.2 ± 0.0 | 0.1 ± 0.0 | 0.1 ± 0.0 | 0.1 ± 0.1 | 0 | 0.1 ± 0.0 | 0.1 ± 0.0 | 0.1 ± 0.0 | 0.3 | 0.1 | 0.1 | 0.1 ± 0.0 | 0.1 ± 0.0 | 0.1 ± 0.0 | 0.1 ± 0.0 | 0.1 ± 0.0 |
| 16:0 (%) | 19.4 ± 0.4 | 19.4 ± 0.5 | 20.2 ± 0.4 | 19.3 ± 0.9 | 20.7 | 20.1 ± 0.6 | 20.5 ± 1.0 | 20.0 ± 0.7 | 19.4 | 20.6 | 19.8 | 19.9 ± 0.4 | 20.5 ± 0.6 | 21.0 ± 1.4 | 21.7 ± 0.0 | 21.2 ± 0.9 |
| 16:1 (%) | 0.3 ± 0.0 | 0.3 ± 0.0 | 0.3 ± 0.0 | 0.2 ± 0.1 | 0.2 | 0.3 ± 0.0 | 0.3 ± 0.0 | 0.3 ± 0.0 | 0.3 | 0.2 | 0.2 | 0.2 ± 0.0 | 0.2 ± 0.0 | 0.2 ± 0.0 | 0.2 ± 0.0 | 0.1 ± 0.0 |
| 16:1n-9 (%) | 0.5 ± 0.1 | 0.5 ± 0.1 | 0.6 ± 0.0 | 0.4 ± 0.1 | 0.3 | 0.6 ± 0.0 | 0.5 ± 0.0 | 0.5 ± 0.1 | 0.4 | 0.6 | 0.4 | 0.3 ± 0.0 | 0.4 ± 0.1 | 0.5 ± 0.0 | 0.6 ± 0.1 | 0.5 ± 0.0 |
| **16:1n-7 (%)** | 3.0 ± 0.4 | 2.5 ± 0.3 | 2.8 ± 0.3 | 2.8 ± 0.3 | 2.8 | 2.6 ± 0.3 | 2.2 ± 0.3 | 2.2 ± 0.8 | 3 | 5 | 2.4 | 2.8 ± 0.4 | 2.5 ± 1.0 | 4.3 ± 1.5 | 4.4 ± 0.5 | 6.4 ± 1.8 |
| 16:1n-5 (%) | 0.4 ± 0.0 | 0.4 ± 0.1 | 0.5 ± 0.0 | 0.4 ± 0.0 | 0.5 | 0.4 ± 0.0 | 0.4 ± 0.0 | 0.5 ± 0.1 | 0.4 | 0.3 | 0.3 | 0.5 ± 0.0 | 0.4 ± 0.1 | 0.3 ± 0.0 | 0.3 ± 0.0 | 0.3 ± 0.1 |
| i-17:0 (%) | 0.7 ± 0.1 | 0.4 ± 0.1 | 0.4 ± 0.0 | 0.5 ± 0.2 | 0.2 | 0.4 ± 0.1 | 0.3 ± 0.0 | 0.3 ± 0.0 | 0.9 | 0.3 | 0.4 | 0.3 ± 0.1 | 0.3 ± 0.1 | 0.4 ± 0.1 | 0.4 ± 0.0 | 0.4 ± 0.0 |
| ai-17:0 (%) | 0.2 ± 0.1 | 0.2 ± 0.0 | 0.1 ± 0.0 | 0.2 ± 0.1 | 0.1 | 0.2 ± 0.0 | 0.1 ± 0.0 | 0.1 ± 0.0 | 0.5 | 0.1 | 0.1 | 0.1 ± 0.0 | 0.1 ± 0.0 | 0.3 ± 0.1 | 0.3 ± 0.0 | 0.3 ± 0.1 |
| 17:0 (%) | 1.1 ± 0.1 | 0.9 ± 0.1 | 0.9 ± 0.0 | 0.9 ± 0.1 | 0.9 | 0.9 ± 0.0 | 0.8 ± 0.0 | 0.8 ± 0.1 | 1.1 | 0.7 | 0.9 | 0.8 ± 0.1 | 0.7 ± 0.1 | 0.7 ± 0.1 | 0.6 ± 0.1 | 0.8 ± 0.3 |
| 17:1 (%) | 0.2 ± 0.0 | 0.3 ± 0.0 | 0.3 ± 0.0 | 0.3 ± 0.0 | 0.3 | 0.3 ± 0.0 | 0.2 ± 0.0 | 0.2 ± 0.0 | 0.4 | 0.3 | 0.3 | 0.3 ± 0.1 | 0.2 ± 0.0 | 0.3 ± 0.1 | 0.3 ± 0.0 | 0.3 ± 0.1 |
| 18:0 (%) | 6.4 ± 0.3 | 6.7 ± 0.5 | 6.2 ± 0.3 | 7.3 ± 0.2 | 7.7 | 6.3 ± 0.3 | 6.6 ± 0.4 | 6.8 ± 0.2 | 7.3 | 6.3 | 7.5 | 7.3 ± 0.1 | 7.3 ± 0.1 | 6.9 ± 0.6 | 6.6 ± 0.2 | 6.1 ± 0.5 |
| 18:1 (%) | 0.0 ± 0.0 | 0.1 ± 0.0 | 0.0 ± 0.0 | 0.1 ± 0.1 | 0 | 0.0 ± 0.0 | 0.0 ± 0.0 | 0.0 ± 0.0 | 0.5 | 0.1 | 0.1 | 0.1 ± 0.0 | 0.1 ± 0.2 | 0.2 ± 0.1 | 0.1 ± 0.0 | 0.2 ± 0.0 |
| **18:1n-9 (%)** | 5.0 ± 0.4 | 5.1 ± 0.5 | 5.5 ± 0.1 | 4.3 ± 0.5 | 3.8 | 5.3 ± 0.4 | 4.7 ± 0.3 | 4.9 ± 0.5 | 3.9 | 6 | 4.5 | 4.3 ± 0.4 | 4.3 ± 0.7 | 6.4 ± 1.6 | 7.4 ± 0.4 | 8.1 ± 1.5 |
| **18:1n-7 (%)** | 4.0 ± 0.2 | 3.3 ± 0.3 | 3.1 ± 0.1 | 4.3 ± 0.7 | 5.2 | 3.0 ± 0.2 | 3.0 ± 0.3 | 3.3 ± 0.5 | 4.3 | 3.6 | 3.5 | 4.4 ± 0.4 | 3.2 ± 0.5 | 3.5 ± 0.2 | 3.0 ± 0.0 | 3.7 ± 0.7 |
| 18:1n-5 (%) | 0.0 ± 0.0 | 0.0 ± 0.0 | 0.0 ± 0.0 | 0.1 ± 0.0 | 0 | 0.0 ± 0.0 | 0.0 ± 0.0 | 0.0 ± 0.0 | 0.1 | 0 | 0 | 0.0 ± 0.0 | 0.1 ± 0.0 | 0.1 ± 0.0 | 0.1 ± 0.0 | 0.1 ± 0.0 |
| 18:2n-6 (%) | 2.4 ± 0.2 | 2.4 ± 0.3 | 2.3 ± 0.1 | 2.4 ± 0.5 | 2 | 2.2 ± 0.1 | 2.2 ± 0.3 | 2.3 ± 0.4 | 2.1 | 2 | 2.5 | 2.4 ± 0.3 | 1.8 ± 0.3 | 2.4 ± 0.3 | 2.2 ± 0.4 | 2.7 ± 0.8 |
| 19:0 (%) | 0.1 ± 0.0 | 0.1 ± 0.0 | 0.1 ± 0.0 | 0.2 ± 0.1 | 0.3 | 0.1 ± 0.0 | 0.1 ± 0.0 | 0.1 ± 0.0 | 0.3 | 0.1 | 0.2 | 0.2 ± 0.0 | 0.1 ± 0.0 | 0.2 ± 0.0 | 0.2 ± 0.0 | 0.2 ± 0.0 |
| 18:3n-6 (%) | 0.2 ± 0.0 | 0.2 ± 0.0 | 0.2 ± 0.0 | 0.2 ± 0.0 | 0.1 | 0.2 ± 0.0 | 0.1 ± 0.0 | 0.1 ± 0.0 | 0.2 | 0.2 | 0.2 | 0.1 ± 0.0 | 0.1 ± 0.0 | 0.2 ± 0.0 | 0.1 ± 0.0 | 0.2 ± 0.0 |
| **18:3n-3 (ALA) (%)** | 3.4 ± 0.6 | 3.3 ± 0.4 | 3.6 ± 0.2 | 2.5 ± 0.5 | 2.1 | 3.4 ± 0.2 | 2.8 ± 0.4 | 2.5 ± 0.7 | 1.7 | 2.1 | 3.3 | 2.0 ± 0.2 | 0.9 ± 0.2 | 1.0 ± 0.2 | 0.9 ± 0.1 | 1.3 ± 0.5 |
| **18:4n-3 (SDA) (%)** | 1.7 ± 0.4 | 1.6 ± 0.5 | 2.1 ± 0.2 | 0.8 ± 0.2 | 0.4 | 1.9 ± 0.2 | 1.4 ± 0.3 | 1.1 ± 0.4 | 0.7 | 1.4 | 0.8 | 0.5 ± 0.2 | 0.3 ± 0.1 | 0.2 ± 0.1 | 0.2 ± 0.1 | 0.3 ± 0.1 |
| 20:0 (%) | 0.2 ± 0.0 | 0.1 ± 0.0 | 0.1 ± 0.0 | 0.2 ± 0.0 | 0.1 | 0.1 ± 0.0 | 0.2 ± 0.0 | 0.1 ± 0.0 | 0.2 | 0.1 | 0.1 | 0.2 ± 0.0 | 0.1 ± 0.0 | 0.2 ± 0.0 | 0.2 ± 0.0 | 0.2 ± 0.0 |
| 20:1n-9 (%) | 0.1 ± 0.0 | 0.1 ± 0.0 | 0.1 ± 0.0 | 0.1 ± 0.0 | 0.1 | 0.1 ± 0.0 | 0.1 ± 0.0 | 0.1 ± 0.0 | 0.1 | 0.2 | 0.1 | 0.1 ± 0.0 | 0.1 ± 0.0 | 0.3 ± 0.1 | 0.3 ± 0.0 | 0.4 ± 0.0 |
| 20:2n-6 (%) | 0.5 ± 0.1 | 0.2 ± 0.0 | 0.1 ± 0.0 | 0.1 ± 0.1 | 0.1 | 0.2 ± 0.0 | 0.1 ± 0.0 | 0.1 ± 0.0 | 0.1 | 0.2 | 0.1 | 0.1 ± 0.0 | 0.1 ± 0.0 | 0.1 ± 0.0 | 0.0 ± 0.0 | 0.0 ± 0.0 |
| **20:4n-6 (ARA) (%)** | 6.4 ± 0.6 | 6.9 ± 0.5 | 7.5 ± 0.3 | 6.3 ± 0.5 | 7.2 | 7.3 ± 0.5 | 9.5 ± 0.8 | 10.0 ± 0.4 | 7.3 | 7.9 | 5.7 | 8.8 ± 0.9 | 9.5 ± 1.9 | 8.7 ± 0.1 | 8.3 ± 0.9 | 7.7 ± 0.6 |
| 20:3n-3 (%) | 0.1 ± 0.0 | 0.1 ± 0.0 | 0.1 ± 0.0 | 0.2 ± 0.1 | 0.1 | 0.1 ± 0.0 | 0.1 ± 0.0 | 0.1 ± 0.0 | 0.3 | 0.1 | 0.2 | 0.1 ± 0.0 | 0.1 ± 0.1 | 0.2 ± 0.0 | 0.1 ± 0.0 | 0.2 ± 0.0 |
| 20:4n-3 (%) | 0.4 ± 0.1 | 0.4 ± 0.1 | 0.5 ± 0.0 | 0.3 ± 0.1 | 0.2 | 0.5 ± 0.0 | 0.4 ± 0.1 | 0.4 ± 0.1 | 0.4 | 0.4 | 0.3 | 0.2 ± 0.0 | 0.2 ± 0.0 | 0.1 ± 0.0 | 0.1 ± 0.0 | 0.1 ± 0.0 |
| **20:5n-3 (EPA) (%)** | 13.3 ± 0.8 | 13.5 ± 0.6 | 15.0 ± 0.4 | 15.4 ± 1.3 | 18.5 | 14.3 ± 0.6 | 16.0 ± 0.7 | 15.5 ± 1.6 | 15.6 | 14.2 | 14.1 | 16.4 ± 1.0 | 13.5 ± 1.8 | 10.9 ± 1.5 | 9.4 ± 1.2 | 10.8 ± 0.1 |
| 22:0 (%) | 0.1 ± 0.0 | 0.1 ± 0.0 | 0.1 ± 0.0 | 0.1 ± 0.0 | 0.1 | 0.1 ± 0.0 | 0.1 ± 0.0 | 0.1 ± 0.0 | 0.1 | 0.1 | 0.1 | 0.1 ± 0.0 | 0.1 ± 0.0 | 0.1 ± 0.0 | 0.1 ± 0.0 | 0.1 ± 0.0 |
| 22:4n-6 (%) | 0.4 ± 0.1 | 0.4 ± 0.0 | 0.5 ± 0.1 | 0.5 ± 0.1 | 0.4 | 0.5 ± 0.0 | 0.5 ± 0.1 | 0.5 ± 0.1 | 0.6 | 0.7 | 0.3 | 0.5 ± 0.0 | 0.6 ± 0.3 | 0.8 ± 0.1 | 0.7 ± 0.2 | 0.9 ± 0.0 |
| 22:5n-6 (%) | 2.4 ± 0.3 | 2.0 ± 0.2 | 1.5 ± 0.1 | 1.4 ± 0.3 | 0.8 | 1.8 ± 0.2 | 1.4 ± 0.2 | 1.5 ± 0.3 | 1.2 | 1.7 | 1.7 | 1.3 ± 0.2 | 1.8 ± 0.4 | 1.7 ± 0.2 | 2.2 ± 0.1 | 1.6 ± 0.5 |
| **22:5n-3 (DPA) (%)** | 1.8 ± 0.2 | 1.9 ± 0.2 | 2.2 ± 0.3 | 3.0 ± 0.7 | 3.8 | 2.0 ± 0.2 | 3.0 ± 0.4 | 3.1 ± 0.2 | 4.9 | 3 | 2.3 | 3.2 ± 0.5 | 3.5 ± 1.2 | 3.0 ± 0.3 | 2.5 ± 0.5 | 3.2 ± 0.1 |
| **22:6n-3 (DHA) (%)** | 22.3 ± 2.6 | 23.3 ± 2.0 | 19.6 ± 0.3 | 22.4 ± 1.4 | 18.9 | 21.5 ± 1.6 | 19.5 ± 1.7 | 19.4 ± 2.7 | 17.9 | 18.3 | 24.9 | 19.9 ± 1.4 | 24.6 ± 3.9 | 22.4 ± 7.0 | 23.9 ± 1.9 | 19.3 ± 3.8 |
| 24:1n-9 (%) | 0.5 ± 0.1 | 0.8 ± 0.1 | 0.7 ± 0.0 | 0.6 ± 0.2 | 0.5 | 0.7 ± 0.1 | 0.7 ± 0.2 | 0.8 ± 0.1 | 0.6 | 0.6 | 0.7 | 0.7 ± 0.2 | 0.8 ± 0.2 | 0.7 ± 0.0 | 0.8 ± 0.1 | 0.6 ± 0.0 |
| ∑SAFAsc (%) | 28.6 ± 0.3 | 28.8 ± 0.4 | 29.3 ± 0.2 | 29.2 ± 0.9 | 30.8 | 29.2 ± 0.5 | 29.6 ± 0.7 | 29.2 ± 0.7 | 29.7 | 29.2 | 29.7 | 29.6 ± 0.3 | 29.5 ± 0.5 | 30.0 ± 1.2 | 30.4 ± 0.0 | 29.7 ± 0.9 |
| ∑MUFAsd (%) | 13.6 ± 0.9 | 12.6 ± 1.0 | 13.2 ± 0.5 | 13.0 ± 1.0 | 13.3 | 12.7 ± 0.9 | 11.5 ± 0.6 | 12.1 ± 1.5 | 13.4 | 16.4 | 11.8 | 13.0 ± 1.2 | 11.4 ± 2.0 | 16.1 ± 3.1 | 16.6 ± 1.1 | 20.1 ± 4.1 |
| ∑PUFAse (%) | 55.2 ± 1.2 | 56.2 ± 1.1 | 55.1 ± 0.4 | 55.6 ± 1.0 | 54.6 | 55.6 ± 0.9 | 57.0 ± 0.8 | 56.7 ± 1.0 | 53 | 52.2 | 56.4 | 55.6 ± 1.0 | 56.9 ± 1.9 | 51.7 ± 4.5 | 50.7 ± 1.1 | 48.2 ± 3.4 |
| n-3f | 43.0 ± 1.5 | 44.1 ± 1.5 | 43.1 ± 0.1 | 44.7 ± 1.3 | 43.9 | 43.6 ± 1.1 | 43.1 ± 1.2 | 42.1 ± 1.7 | 41.5 | 39.5 | 46 | 42.3 ± 1.8 | 43.1 ± 1.7 | 37.9 ± 4.9 | 37.2 ± 0.3 | 35.1 ± 3.2 |
| n-6g | 12.2 ± 0.8 | 12.1 ± 0.6 | 12.0 ± 0.3 | 10.9 ± 1.0 | 10.7 | 12.0 ± 0.5 | 13.8 ± 0.9 | 14.7 ± 0.9 | 11.5 | 12.8 | 10.4 | 13.3 ± 1.2 | 13.8 ± 2.1 | 13.8 ± 0.4 | 13.5 ± 1.4 | 13.1 ± 0.3 |
| n-3/n-6 | 3.5 ± 0.3 | 3.7 ± 0.3 | 3.6 ± 0.1 | 4.1 ± 0.5 | 4.1 | 3.6 ± 0.2 | 3.1 ± 0.3 | 2.9 ± 0.3 | 3.6 | 3.1 | 4.4 | 3.2 ± 0.4 | 3.2 ± 0.5 | 2.8 ± 0.4 | 2.8 ± 0.3 | 2.7 ± 0.2 |

a Number of perch for each group, b total length, c saturated fatty acids, d monounsaturated fatty acids, e polyunsaturated fatty acids, f omega-3 PUFAs, g omega-6 PUFAs.

# References

Bartels P, Hirsch PE, Svanbäck R, Eklöv P (2016) Dissolved organic carbon reduces habitat coupling by top predators in lake ecosystems. Ecosystems 19:955–967. https://doi.org/10.1007/s10021-016-9978-x

Caut S, Angulo E, Courchamp F (2008) Caution on isotopic model use for analyses of consumer diet. Can J Zool 86:438–445. https://doi.org/10.1139/Z08-012

Christie WW (2018) Mass spectrometry of methyl ester derivatives of fatty acids. [http://www.lipidhome.co.uk/ms/methylesters.htm.](http://www.lipidhome.co.uk/ms/methylesters.htm) Accessed 20 May 2016

Clarke KR, Gorley RN, Somerfield PJ (2014) Change in marine communities: an approach to statistical analysis and interpretation, 3rd edn. PRIMER-E Ltd, Plymouth, UK

Eklöv P (1997) Effects of habitat complexity and prey abundance on the spatial and temporal distributions of perch (*Perca fluviatilis*) and pike (*Esox lucius*). Can J Fish Aquat Sci 54:1520–1531. https://doi.org/10.1139/f97-059

Gannes LZ, O’Brien DM, Martinez del Rio C (1997) Stable Isotopes in Animal Ecology: Assumptions, Caveats, and a Call for More Laboratory Experiments. Ecology 78:1271–1276. https://doi.org/10.1890/0012-9658(1997)078[1271:SIIAEA]2.0.CO;2

Hopkins JB III, Ferguson JM (2012) Estimating the diets of animals using stable isotopes and a comprehensive Bayesian mixing model. PLoS ONE 7:e28478. https://doi.org/10.1371/journal.pone.0028478

Marklund MHK, Svanbäck R, Faulks L, et al (2019) Asymmetrical habitat coupling of an aquatic predator—The importance of individual specialization. Ecol Evol 9:3405–3415. https://doi.org/10.1002/ece3.4973

McCutchan JH, Lewis WM, Kendall C, McGrath CC (2003) Variation in trophic shift for stable isotope ratios of carbon, nitrogen, and sulfur. Oikos 102:378–390. https://doi.org/10.1034/j.1600-0706.2003.12098.x

Moore JW, Semmens BX (2008) Incorporating uncertainty and prior information into stable isotope mixing models. Ecol Lett 11:470–480

Ogle DH (2017) FSA: Fisheries Stock Analysis. R package

Parnell AC, Phillips DL, Bearhop S, et al (2013) Bayesian stable isotope mixing models. Environmetrics 24:387–399. https://doi.org/10.1002/env.2221

Persson L (1983) Food consumption and competition between age classes in a perch *Perca fluviatilis* population in a shallow eutrophic lake. Oikos 40:197–207. https://doi.org/10.2307/3544583

Persson L, Bystrom P, Wahlstrom E (2000) Cannibalism and competition in Eurasian perch: population dynamics of an ontogenetic omnivore. Ecology 81:1058–1071. https://doi.org/10.2307/177178

Phillips DL, Inger R, Bearhop S, et al (2014) Best practices for use of stable isotope mixing models in food-web studies. Can J Zool 92:823–835. https://doi.org/10.1139/cjz-2014-0127

Post DM (2002) Using stable isotopes to estimate trophic position: models, methods, and assumptions. Ecology 83:703–718. https://doi.org/10.1890/0012- 9658(2002)083[0703:USITET]2.0.CO;2

R Core Team (2019) R: A language and environment for statistical computing. R Foundation for Statistical Computing, Vienna, Austria

Scharnweber K, Strandberg U, Marklund MHK, Eklöv P (2016) Combining resource use assessment techniques reveals trade-offs in trophic specialization of polymorphic perch. Ecosphere 7:e01387. https://doi.org/10.1002/ecs2.1387

Semmens, B. X. et al. 2009. Quantifying inter- and intra-population niche variability using hierarchical Bayesian stable isotope mixing models. - PLoS ONE 4: e6187.

Stock BC, Semmens BX (2016a) MixSIAR GUI user manual, version 3.1

Stock BC, Semmens BX (2016b) Unifying error structures in commonly used biotracer mixing models. Ecology 97:2562–2569. https://doi.org/10.1002/ecy.1517

Svanbäck R, Eklöv P (2003) Morphology dependent foraging efficiency in perch: a trade-off for ecological specialization? Oikos 102:273–284. https://doi.org/10.1034/j.1600- 0706.2003.12657.x

Ward EJ, Semmens BX, Schindler DE (2010) Including source uncertainty and prior information in the analysis of stable isotope mixing models. Environ Sci Technol 44:4645– 4650. https://doi.org/10.1021/es100053v

Wolf N, Carleton SA, Martinez del Rio C (2009) Ten years of experimental animal isotopic ecology. Funct Ecol 23:17–26. https://doi.org/10.1111/j.1365-2435.2009.01529.x
